# Synthesis of 1-indanones with a broad range of biological activity

**DOI:** 10.3762/bjoc.13.48

**Published:** 2017-03-09

**Authors:** Marika Turek, Dorota Szczęsna, Marek Koprowski, Piotr Bałczewski

**Affiliations:** 1 Institute of Chemistry, Environmental Protection and Biotechnology, The Faculty of Mathematics and Natural Sciences, Jan Długosz University in Częstochowa, Armii Krajowej 13/15, Częstochowa, 42-201, Poland; 2 Department of Structural Biology, Faculty of Biomedical Sciences and Postgraduate Education, Medical University of Łódź, Żeligowskiego 7/9, 90-752, Łódź, Poland; 3 Department of Heteroorganic Chemistry, Centre of Molecular and Macromolecular Studies, Polish Academy of Sciences, Sienkiewicza 112, Łódź, 90-363, Poland

**Keywords:** biological activity, Diels–Alder reaction, Friedel–Crafts reaction, 1-indanones, Nazarov reaction

## Abstract

This comprehensive review describes methods for the preparation of 1-indanones published in original and patent literature from 1926 to 2017. More than 100 synthetic methods utilizing carboxylic acids, esters, diesters, acid chlorides, ketones, alkynes, alcohols etc. as starting materials, have been performed. This review also covers the most important studies on the biological activity of 1-indanones and their derivatives which are potent antiviral, anti-inflammatory, analgesic, antimalarial, antibacterial and anticancer compounds. Moreover, they can be used in the treatment of neurodegenerative diseases and as effective insecticides, fungicides and herbicides.

## Introduction

**Figure 1:**
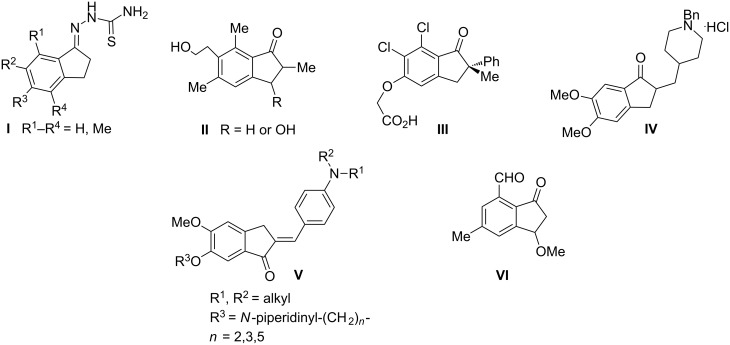
Biologically active 1-indanones and their structural analogues.

In the last few years, 1-indanone derivatives and their structural analogues have been widely used in medicine, agriculture and in natural products synthesis [1–3]. In addition, structurally related indanes also showed biological activity and have been reviewed by Ahmed in 2016 [4]. Extensive studies on bioactivity of 1-indanone derivatives open up more and more new possibilities of their applications as antiviral and antibacterial agents [5] (**I** and **II**), anticancer drugs [6] (**VI**), pharmaceuticals used in the Alzheimer’s disease treatment [7] (**III**), cardiovascular drugs [7] (**IV**), insecticides, fungicides, herbicides [8] (**V**) and non-nucleoside, low molecular drugs for the hepatitis C treatment, which inhibit HCV replication [9–10] (Figure 1).

**Figure 2:**
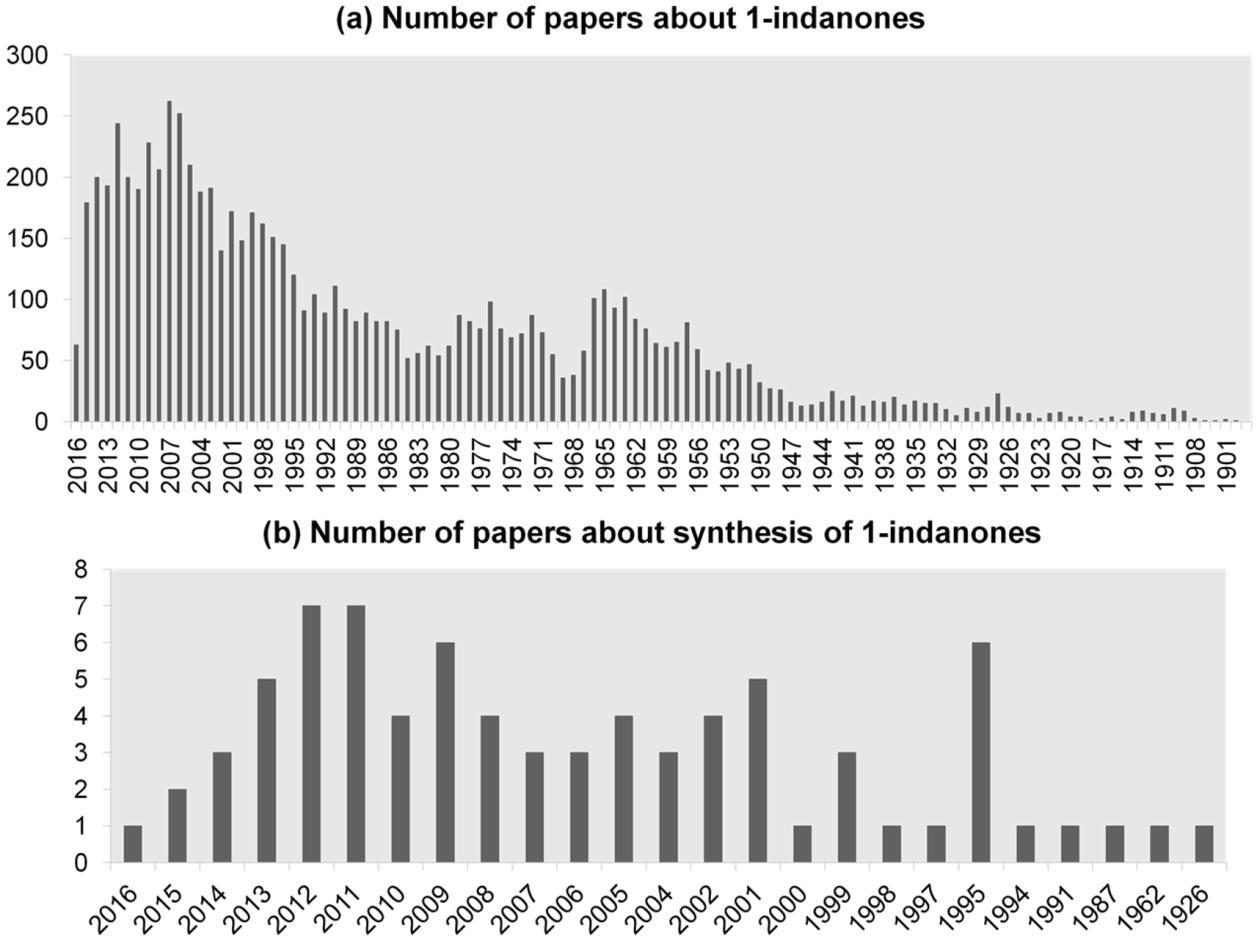
Number of papers about (a) 1-indanones, (b) synthesis of 1-indanones.

First publications concerning the preparation of 1-indanones appeared in the 1920s and since then this field has been intensively developed [11]. A huge interest in 1-indanones and their derivatives resulted in a considerable number of papers concerning their synthesis (Figure 2). The commonly used reaction in this area is the Nazarov reaction which employs α,β-unsaturated ketones as substrates and is carried out in the presence of Brønsted or Lewis acids. Despite extensive studies on 1-indanones and their biological activity, this group of compounds has never been reviewed in literature and therefore the present work is the first, comprehensive review of synthetic methods and applications of these compounds in medicine and agriculture published from 1926 to 2017.

We have divided the review into 4 sections, taking into account the formation of the 5- (section 1), 6- (section 2) and simultaneous formation of 5- and 6-membered rings of 1-indanones (section 3) as well as functionalization of 1-indanones or related compounds (section 4).

## Review

### 1 Construction of the 5-membered ring

#### 1.1 From carbonyl compounds

**Scheme 1:**
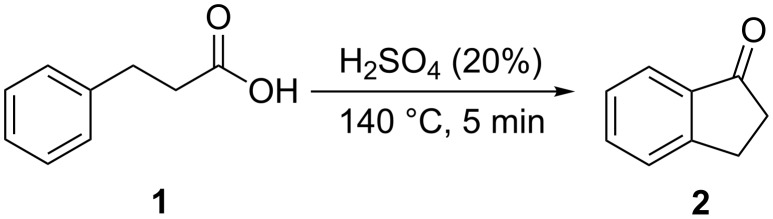
Synthesis of 1-indanone (**2**) from hydrocinnamic acid (**1**).

**1.1.1 From carboxylic acids:** The first synthesis of 1-indanone from carboxylic acid has been described by Price and Lewis in 1939 [12]. They cyclized hydrocinnamic acid (**1**) to the unsubstituted 1-indanone (**2**), in 27% yield using 20% sulfuric acid at 140 °C (Scheme 1).

**Scheme 2:**
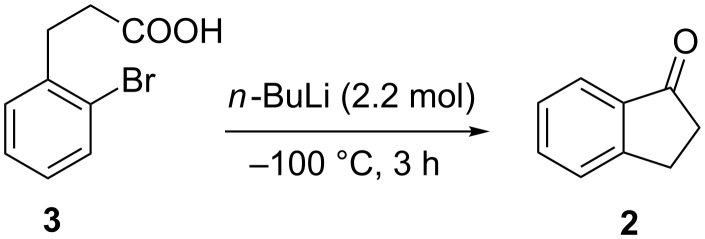
Synthesis of 1-indanone (**2**) from 3-(2-bromophenyl)propionic acid (**3**).

Another reaction leading to the formation of the unsubstituted 1-indanone (**2**) in higher 76% yield utilizes the cyclization of 3-(2-bromophenyl)propionic acid (**3**), conducted at −100 °C in the presence of *n*-BuLi (Scheme 2) [13].

**Scheme 3:**
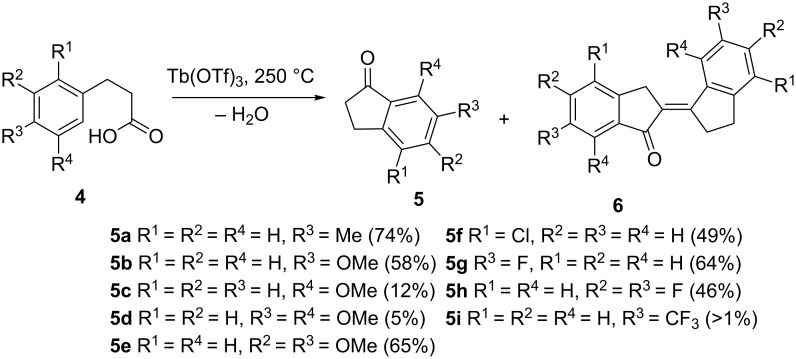
Synthesis of 1-indanones **5** from 3-arylpropionic acids **4**.

A cyclization of 3-arylpropionic acids **4**, catalyzed by Tb(OTf)_3_ at 250 °C, led to the formation of at the aryl ring substituted 1-indanones **5** in yields of up to 74% and trace amounts of the auto-condensation products **6** (Scheme 3). Even in the deactivated derivatives containing halogen atoms at the aromatic system, the cyclopentanone ring closure took place quite easily [14]. If other Lewis acids, such as Bi(NTf_2_)_3_ or triflate derivatives of the transition metals (In, Sc, Ce, Pr, Nd, Eu, Dy, Yb, Lu, Hf, Gd) were used, the cyclization proceeded in unsatisfactory yields and with a large number of unidentified byproducts.

**Scheme 4:**
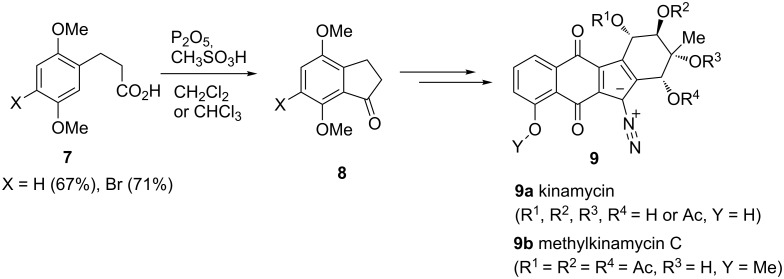
Synthesis of kinamycin (**9a**) and methylkinamycin C (**9b**).

Cyclization of other 3-arylpropionic acids **7** led to the formation of 4,7-dimethoxy-1-indanones **8**. They were used in the key step of the synthesis of kinamycin **9** derivatives, which exhibited a strong cytotoxic and anticancer activity (Scheme 4) [15].

**Scheme 5:**
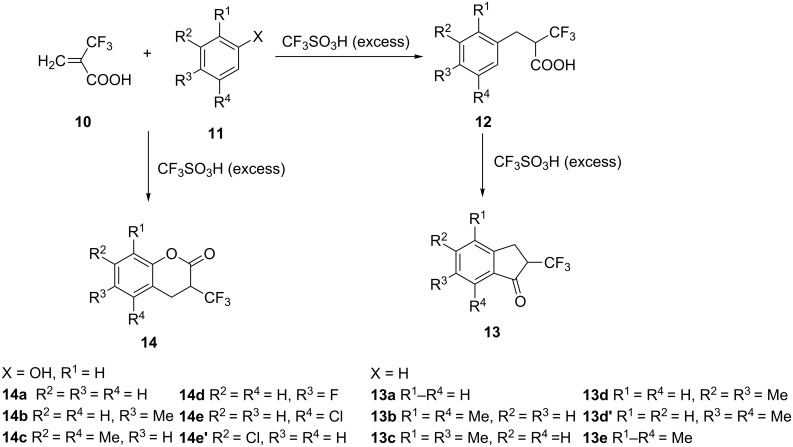
Synthesis of trifluoromethyl-substituted arylpropionic acids **12**, 1-indanones **13** and dihydrocoumarins **14**.

Propionic acid derivatives are also useful substrates in syntheses of 1-indanones and isocoumarins [16]. These latter are essential reagents for the synthesis of bioactive compounds. Fluoroorganic compounds play a significant role as very effective therapeutics, and for this reason, Prakash, Olah et al. synthesized in 2010 trifluoromethyl-substituted arylpropanoic acids **12**, 1-indanones **13** and dihydrocoumarins **14** (Scheme 5) [17]. These products have been obtained by utilizing arenes/phenols **11** (X = H/OH) and 2-(trifluoromethyl)acrylic acid (**10**) as a result of a Friedel–Crafts alkylation.

**Scheme 6:**
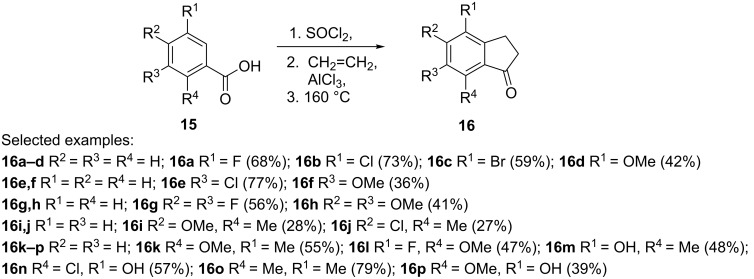
Synthesis of 1-indanones **16** from benzoic acids **15**.

An efficient and scalable one-pot process for the preparation of 1-indanones from benzoic acids has been described by Huang et al. [18]. In this synthesis, acyl chlorides formed in the reaction of benzoic acids **15** with thionyl chloride, reacted with ethylene and the resulting intermediates underwent an intramolecular Friedel–Crafts alkylation to form 1-indanones **16** (Scheme 6).

**Scheme 7:**
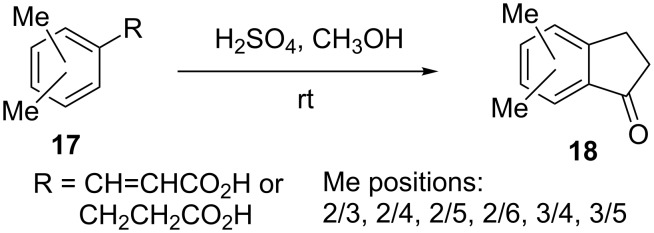
Synthesis of 1-indanones **18** from arylpropionic and 3-arylacrylic acids **17**.

Both arylpropionic and 3-arylacrylic acids **17** underwent cyclization in the presence of polyphosphoric and sulfuric acids to form 1-indanones **18** in good yields (60–90%) (Scheme 7) [19].

**Scheme 8:**
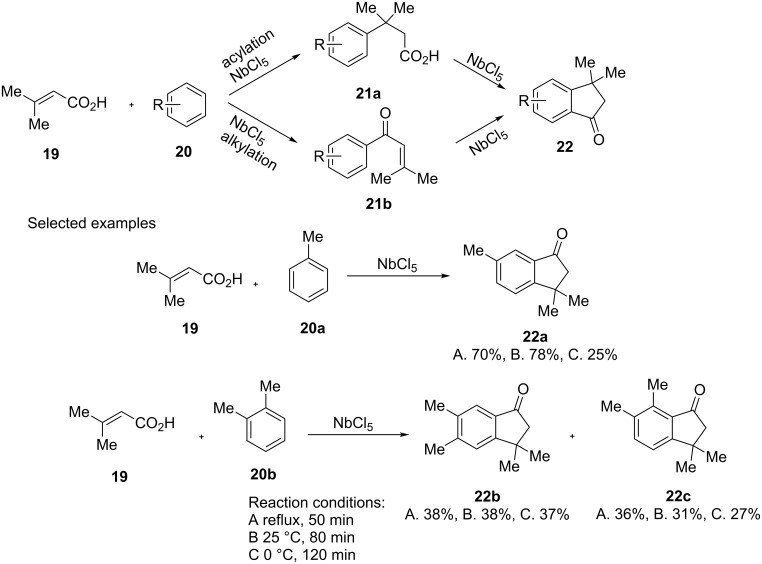
The NbCl_5_-induced one-step synthesis of 1-indanones **22**.

A one-step synthesis of 1-indanones **22** through the NbCl_5_-induced Friedel–Crafts reaction, has been described by Barbosa et al. in 2015 [20]. The reaction was carried out using 3,3-dimethylacrylic acid (**19**), aromatic substrate **20** and highly oxophilic NbCl_5_ as a catalyst. By varying the type of substrate, a variety of 1-indanone derivatives **22** was obtained. Depending on the reaction conditions (A–C), 1-indanone derivatives **22** was obtained in 0–78% yields. The studies indicated that of two possible intermediates **21a** and **21b**, obtained as a result of acylation or alkylation reaction, the intermediate **21a** with activated aromatic ring, always led to the 1-indanone formation, in contrast to the acylated intermediate **21b** with deactivated aromatic ring (Scheme 8).

In the same year, Xu et al. have patented a synthesis of 5-chloro-1-indanone via the reaction of malonic acid with chlorobenzaldehyde [21]. In the first step, the substrates reacted in the presence of formic acid and diethylamine to form 3-chlorophenylpropionic acid followed by a intramolecular Friedel–Crafts acylation with malonyl chloride in the presence of zinc chloride to give 5-chloro-1-indanone.

**Scheme 9:**
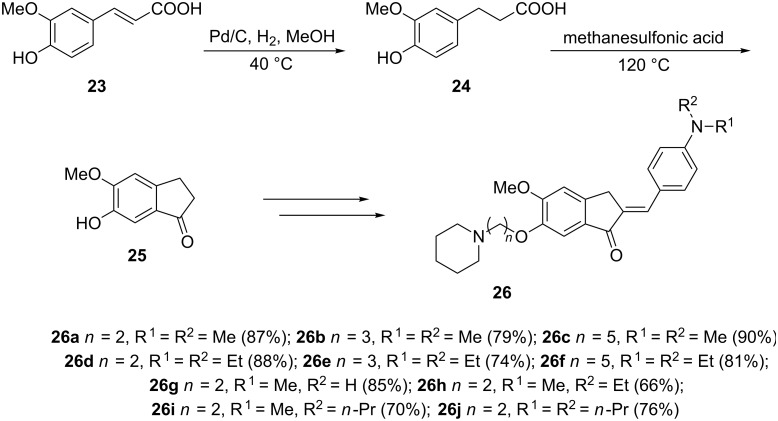
Synthesis of biologically active 1-indanone derivatives **26**.

New 1-indanone derivatives that may be used as multi-functional drugs for the treatment of Alzheimer's disease have been synthesized by Li et al. [8]. In this synthesis, ferulic acid (**23**) was hydrogenated in the presence of Pd/C catalyst to give the saturated derivative **24** and then cyclized to the 1-indanone **25**. The latter was then converted to biologically active 1-indanone derivatives **26** in three steps (Scheme 9). The authors tested activities of the synthesized compounds **26** for inhibition of cholinesterases (AChE and BuChE) and inhibition of amyloid beta (Aβ) self-assembly. The studies have shown that most of the compounds **26** exhibited a good inhibitory activity against AChE. For instance, compounds **26d** and **26i** demonstrated IC_50_ values of 14.8 and 18.6 nM, respectively and a remarkably inhibition of Aβ aggregation.

The environmentally benign synthesis of 1-indanones from 3-arylpropanoic and 4-arylbutanoic acids has been reported in 2015 by Le et al. [22]. The authors applied a microwave-assisted intramolecular Friedel–Crafts acylation catalyzed by metal triflate in triflate-anion containing ionic liquids. This synthesis proceeded with the goals of green chemistry and allowed to obtain 1-indanones in good yields. Moreover, the metal triflate could be recovered and reused without loss of catalytic activity.

**Scheme 10:**
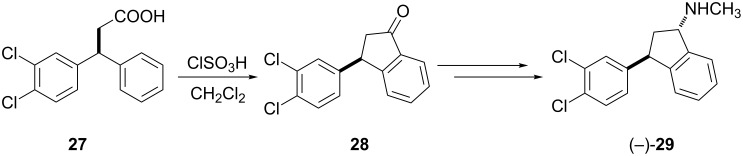
Synthesis of enantiomerically pure indatraline ((−)-**29**).

Indatraline that blocks the action of cocaine contains moieties having antidepressant, antihistamine and blood pressure-lowering properties. Yun et al. [23] have developed a new method for the synthesis of pure indatraline ((−)-**29**) in a sequence of reactions starting from carboxylic acid **27** (Scheme 10).

**1.1.2 From acid chlorides:** The first synthesis of unsubstituted 1-indanone (**2**), obtained from the reaction of phenylpropionic acid chloride with aluminum chloride in benzene (90% yield), has been published in 1927 [24]. In the same year, Mayer and Müller have described a cyclization of unsaturated ketones with acid chlorides leading to the formation of 1-indanones [25].

**Scheme 11:**
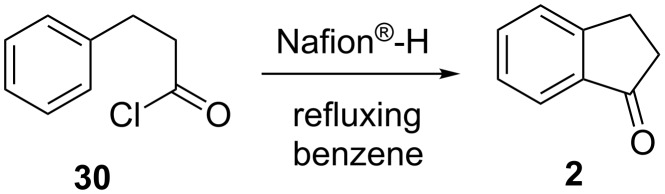
Synthesis of 1-indanone (**2**) from the acyl chloride **30**.

The use of other acidic catalysts, like ZnBr_2_ or Nafion^®^-H also led to the formation of 1-indanones [26–27]. Thus, treatment of the acid chloride **30** with Nafion^®^-H in refluxing benzene gave unsubstituted 1-indanone (**2**) in 90% yield (Scheme 11). In the reaction described above, acid chloride groups reacted with free sulfonic acid groups of Nafion^®^-H to generate in situ highly reactive mixed anhydrides which cyclized to produce cyclic ketones. In order to complete the catalytic cycle, Nafion^®^-H was regenerated in the acylation step.

**Scheme 12:**
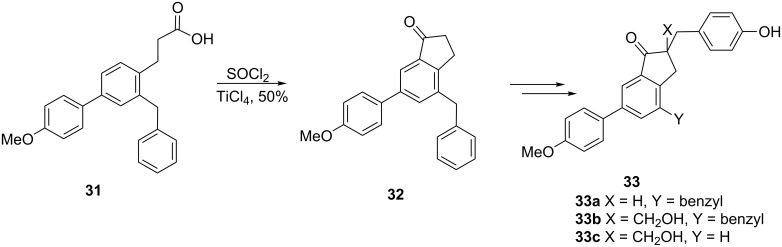
Synthesis of the mechanism-based inhibitors **33** of coelenterazine.

Investigation of luminescence is a source of valuable information in modern molecular biology, immunology and embryology. An example of a bioluminescent molecule is coelenterazine (luciferin) of which three inhibitors containing an 1-indanone core **33** have been synthesized to follow the bioluminescence reaction mechanism [28]. The intramolecular Friedel–Crafts acylation of 3-arylpropionic acid derivative **31** followed by conversion of the acid to the corresponding acyl chloride with thionyl chloride, led to the formation of 1-indanone **32** which was further transformed into the desired inhibitors **33** (Scheme 12).

In the synthesis of 5-hydroxy-1-indanone, Chen and Li reacted 3-chloropropionyl chloride with 2,6-dibromophenol to give 2,6-dibromophenyl 3-chloropropionate [29]. Next, the latter was converted to 4,6-dibromo-5-hydroxy-1-indanone in the presence of a Lewis acid and then transformed to 5-hydroxy-1-indanone as a result of debromination.

**Scheme 13:**
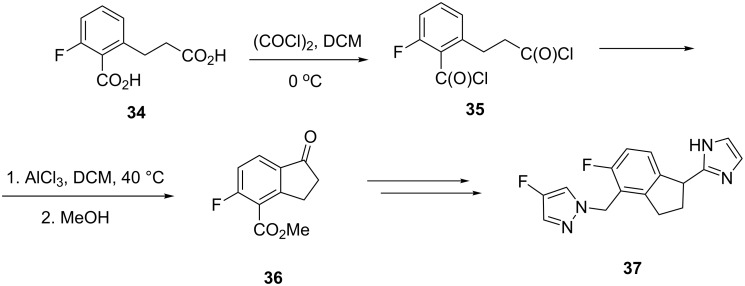
Synthesis of the indane 2-imidazole derivative **37**.

Adrenergic receptors are metabotropic receptors located on cell membranes and stimulated by catecholamines, especially adrenaline and noradrenaline. A new method for the synthesis of the indane 2-imidazole derivative **37** acting as a strong adrenergic receptor agonist has been proposed by Roberts et al. [30]. In this synthesis, the diacid **34** was converted to 1-indanone **36** via the AlCl_3_ promoted Friedel–Crafts acylation of the acid dichloride **35**. Then, in a sequence of reactions, the 1-indanone **36** was transformed to **37** (Scheme 13).

**Scheme 14:**
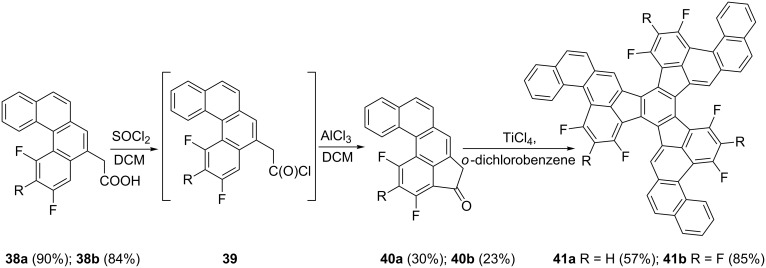
Synthesis of fluorinated PAHs **41**.

Kabdulov, Amsharov and Jansen have proposed a methodology for the synthesis of fluorinated polyaromatic hydrocarbons (PAH’s) via the 1-indanone intermediates **40** [31]. In this synthesis, acids **38** have been transformed to the corresponding acid chlorides **39**, followed by an intramolecular Friedel–Crafts acylation in the presence of AlCl_3_ in dichloromethane to give the corresponding 1-indanones **40**. The latter were cyclized using TiCl_4_ in *o*-dichlorobenzene to fluorinated PAHs **41** (Scheme 14).

**1.1.3 From esters and diesters:** In 1951, Gilmore has demonstrated that use of esters, rather than free arylpropionic acids in phosphoric acid, in the presence of phosphorus pentoxide also led to 1-indanones in equally good or even better yields [32].

**Scheme 15:**
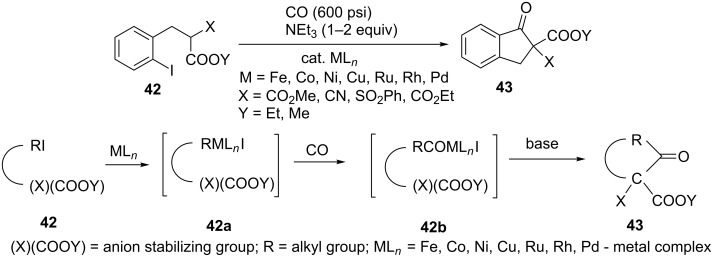
Synthesis of 1-indanones **43** via transition metal complexes-catalyzed carbonylative cyclization of methyl and ethyl esters **42** and a general mechanism of this reaction.

Transition metal complexes have been used by Negishi et al. as catalysts in the carbonylative cyclization reaction of carboxylic acid methyl and ethyl esters **42** which led to the formation of 1-indanones **43** [33]. This reaction was carried out in acetonitrile, in the presence of triethylamine, under carbon monoxide atmosphere, achieving efficiency in the range of 88–92%, when using lithium, nickel and palladium catalysts (Scheme 15). A general mechanism illustrating the role of transition metal complexes and CO in this reaction is shown in Scheme 15.

Cyclic esters were also used in the syntheses of 1-indanones. Thus, by adding β-propiolactone to aluminum chloride in benzene, 1-indanone has been obtained in 80% yield. Interestingly, when aluminum chloride was added to the lactone in benzene, the yield of this reaction decreased (30%) [34].

**Scheme 16:**
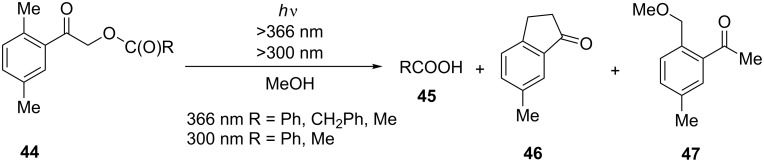
Synthesis of 6-methyl-1-indanone (**46**).

Irradiation of esters **44** possessing the photoremovable 2,5-dimethylphenacyl group in benzene or cyclohexane solutions led to free carboxylic acids **45** (85–95%) accompanied by 6-methyl-1-indanone (**46**) which was formed as a byproduct in 5–15% yields [35]. Irradiation of esters **44** in methanol gave 6-methyl-1-indanone (**46**) along with 2-(methoxymethyl)-5-methylacetophenone (**47**) and the corresponding free carboxylic acid **45** (Scheme 16).

**Scheme 17:**
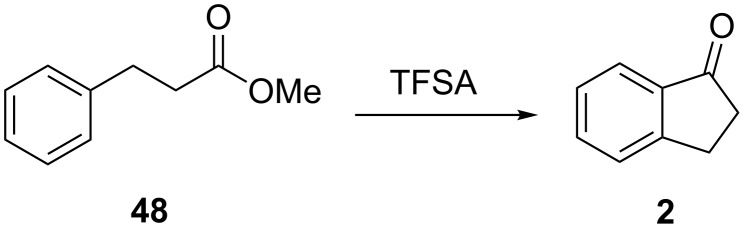
Synthesis of 1-indanone (**2**) from ester **48**.

The unsubstituted 1-indanone (**2**) has been synthesized quantitatively by Nakamura, Sugimoto and Ohwada via trifluoromethanesulfonic acid (TFSA)-catalyzed intramolecular cyclization of ester **48** (Scheme 17) [36].

In 2013, Zhou and Matsuya have proposed an effective method for the preparation of 5,7-dimethoxy-1-indanone [37]. In this synthesis, a mixture of diethyl 2-(3,5-dimethoxybenzyl)malonate and methanesulfonic acid was stirred at 100 °C for 2 h and 5,7-dimethoxy-1-indanone was obtained in excellent yield (95%).

**Scheme 18:**
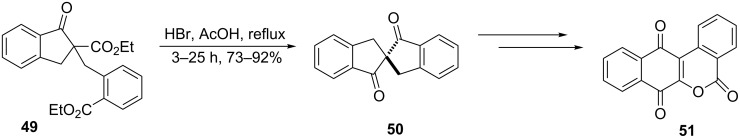
Synthesis of benzopyronaphthoquinone **51** from the spiro-1-indanone **50**.

A new route for the synthesis of an anticancer agent, benzopyronaphthoquinone **51** from the spiroindanone **50** has been proposed by Estévez et al. [38]. Thus, starting from 2,2-disubstituted-1-indanone **49**, the spiro-1-indanone **50** was formed via cyclization using HBr/AcOH and next converted in a sequence of reactions to the biologically active benzopyronaphthoquinone **51** (Scheme 18).

**Scheme 19:**
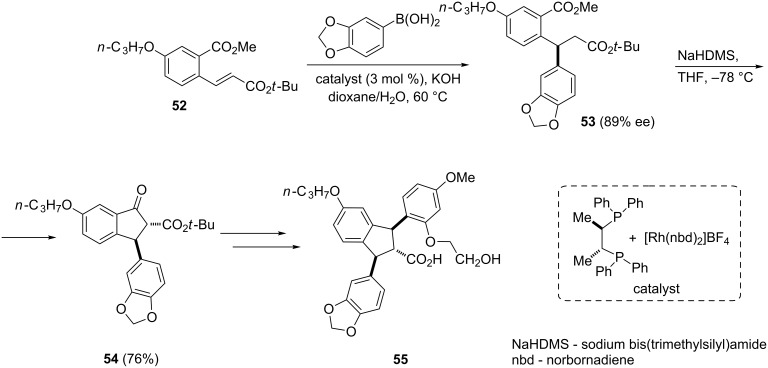
Synthesis of the selective endothelin A receptor antagonist **55**.

Endothelins are 21-amino acid peptides with vasoconstrictor properties, produced primarily in the endothelium. They play a key role in vascular homeostasis and are responsible for proper vascular tone and vascular perfusion maintaining. In 2006, Miyaura et al. have synthesized selective endothelin A receptor antagonists **55** via a formal 1,4-addition of arylboronic acids to β-aryl-α,β-unsaturated ketones and esters [39]. Thus, the α,β-unsaturated diester **52** was coupled with arylboronic acid in the presence of rhodium(I)/Chiraphos^®^ complex as a catalyst to obtain derivative **53**, which next underwent a Claisen condensation to form 1-indanone **54**. The latter was further used as a substrate for the synthesis of selective endothelin A receptor antagonist **55** (Scheme 19).

**Scheme 20:**
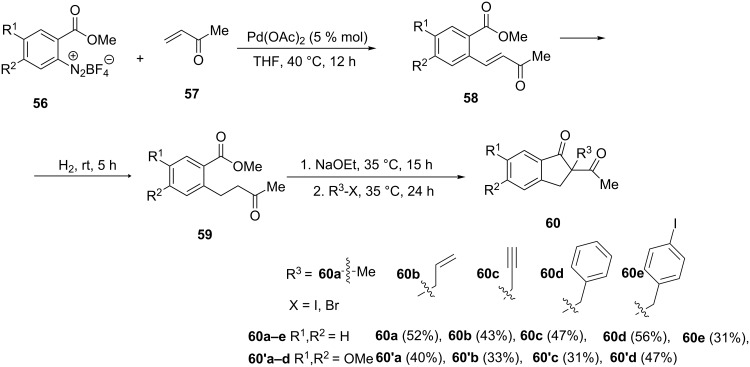
Synthesis of 1-indanones **60** from methyl vinyl ketone (**57**).

A simple and efficient synthesis of 1-indanones **60** from methyl vinyl ketone (**57**) has been proposed by Felpin et al. [40]. In this synthesis, the authors have applied a Heck-reduction–cyclization–alkylation (HRCA) methodology under mild and simple reaction conditions. First, diazonium salts **56** underwent the Heck reaction with methyl vinyl ketone (**57**) to give the cross-coupling products **58** followed by hydrogenation of the latter to give aromatic ketoesters **59**. The base-mediated cyclization of the latter in the presence of sodium ethoxide led to the formation of the corresponding 1-indanone anions α to carbonyl, which next were alkylated to give 2-substituted 1-indanones **60** (Scheme 20). This one-pot process utilizing a multi-task palladium catalyst allowed the synthesis of **60** in yields ranged from 31 to 56%.

**Scheme 21:**
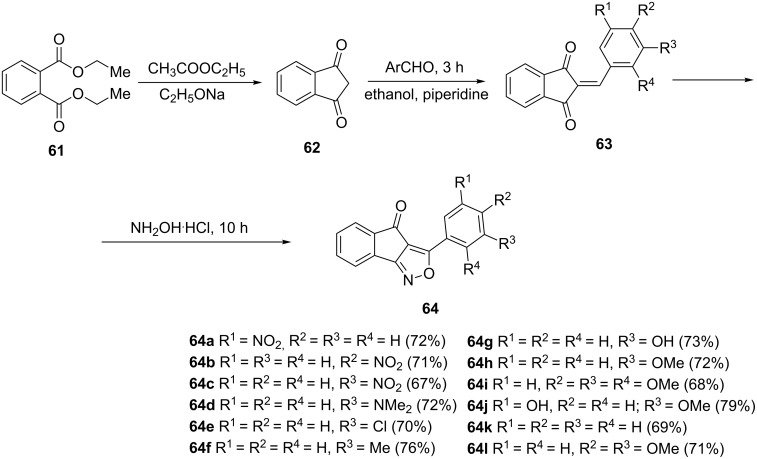
Synthesis of 1-indanones **64** from diethyl phthalate **61**.

1-Indanones exhibit a broad spectrum of biological activity including anti-inflammatory [41], analgesic [42], antimicrobial [43], antiviral [5], anticancer [44] and antimalarial [45] activity. A combination of two or more biologically active moieties may increase or decrease the biological activity. A series of isoxazole fused 1-indanones **64** with increased anti-inflammatory and antimicrobial activity has been synthesized by Giles et al. [46]. In this synthesis, diethyl phthalate (**61**) was reacted with ethyl acetate to obtain indane-1,3-dione (**62**), followed by a Knoevenagel condensation with a variety aromatic aldehydes to give chalcone derivatives **63** (Scheme 21). The reaction of the latter with hydroxylamine hydrochloride, followed by intramolecular 1,4-addition gave 1-indanone derivatives **64a–l** which were further tested for in vitro antibacterial activity against *Escherichia coli* and *Bacillus subtilis*, and antifungal activity against *Aspergillus niger* and *Penicillium notatum*. Among the synthesized series of 1-indanone derivatives **64**, the highest antibacterial activity was exhibited by derivatives **64k** and **64l**, whereas the most potent antifungal activity was revealed for derivatives **64h** and **64j**. The authors have also studied anti-inflammatory properties of these derivatives using the carrageenan induced paw edema method in rats. The anti-inflammatory activity of the synthesized compounds was compared with standard indomethacin (a non-steroidal anti-inflammatory drug used in rheumatoid arthritis treatment). 1-Indanone derivatives **64k**, **64j**, **64f**, **64g** and **64i** exhibited a stronger inhibition of the paw edema than indomethacin.

**Scheme 22:**
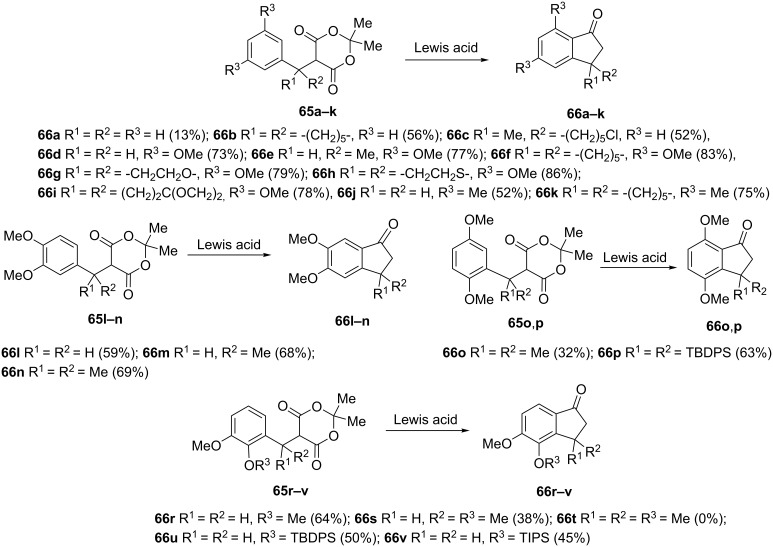
Synthesis of 1-indanone derivatives **66** from various Meldrum’s acids **65**.

The use of Meldrum’s acids **65** is an alternative method for the synthesis of 1-indanones via intramolecular Friedel–Crafts reaction. These compounds are stable at room temperature, easily prepared, functionalized, handled and purified. Thus, the intramolecular Friedel–Crafts acylation of aromatics with Meldrum's acid derivatives **65,** catalyzed by metal trifluoromethanesulfonates such as Sc(OTf)_3_, Dy(OTf)_3_, Yb(OTf)_3_, has been reported in 2005 [6]. The studied Meldrum's acid derivatives **65** were functionalized at α- and/or β-positions by alkyl, haloalkyl, alkenyl, alkynyl, nitrile, ether, thioether, triisopropylsilyl (TIPS) or *tert*-butyldiphenylsilyl (TBDPS) groups. Depending on the type of catalyst and substrate, 1-indanone derivatives **66** were obtained in 13–86% yields (Scheme 22). This method was further applied to the synthesis of biologically active compounds, such as 1-tetralones, 1-benzosuberones and donepezil (a potent acetylcholinesterase inhibitor used in the treatment of Alzheimer’s disease).

**Scheme 23:**
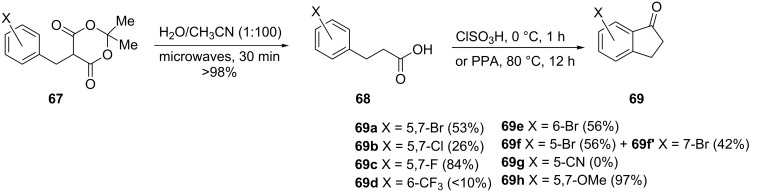
Synthesis of halo 1-indanones **69**.

Halo-1-indanones **69** were synthesized from benzyl Meldrum’s acids derivatives **67** in two steps [47]. In this synthesis, they underwent microwave-assisted hydrolysis to carboxylic acids **68**, followed by chlorosulfonic acid-mediated Friedel–Crafts cyclization to give halo-1-indanones **69** (Scheme 23).

**Scheme 24:**
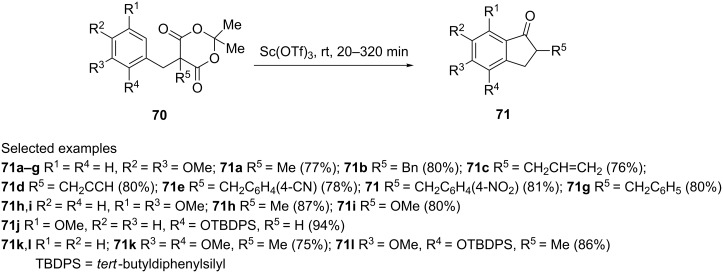
Synthesis of substituted 1-indanones **71**.

Quaternized Meldrum’s acids **70** have been used for the synthesis of 1-indanones **71** [48]. The reaction was catalyzed by Sc(OTf)_3_ and proceeded in very good yields (up to 94%) (Scheme 24).

**Scheme 25:**
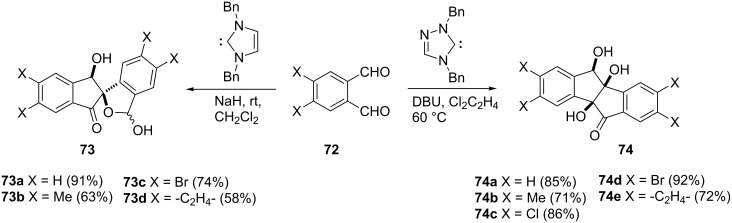
Synthesis of spiro- and fused 1-indanones **73** and **74**.

**1.1.4 From aldehydes and dialdehydes:** The stereoselective dimerization reaction of phthalaldehydes **72**, catalyzed by a *N*-heterocyclic carbene is an outstanding protocol for the synthesis of polyhydroxylated spiro- or fused 1-indanones [49]. Thus, the imidazole-based carbene catalyzed the conversion of phthalaldehydes **72** to dihydroxyspiro[indane-2,1′-isobenzofuran]-3-ones **73**, whereas triazole-based carbene catalyzed the conversion of **72** to *cis*-trihydroxyindano[2,1-*a*]indan-5-ones **74** (Scheme 25).

**Scheme 26:**
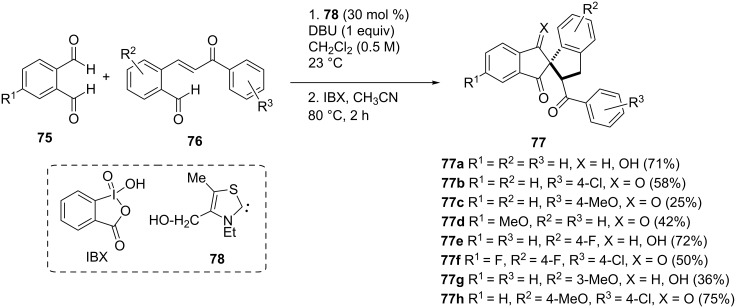
Synthesis of spiro-1,3-indanodiones **77**.

**Scheme 27:**
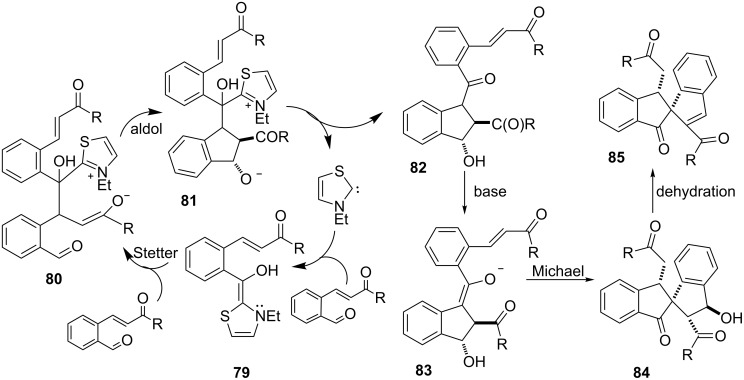
Mechanistic pathway for the NHC-catalyzed Stetter–Aldol–Michael reaction.

Another example of the 1-indanone synthesis using *N*-heterocyclic carbenes (NHC) has been described by Gravel et al. [50–51]. The benefit of the described reaction was a rapid construction of three new carbon–carbon bonds and a carbon quaternary center with high diastereoselectivity as a consequence of the Stetter–Aldol–Aldol (SAA) reaction sequence. The Stetter–Aldol–Aldol conversion of the phthaldialdehyde derivatives **75** and *o*-formyl substituted chalcones **76** using the thiazole based carbene **78** as a precatalyst allowed to obtain spiro-1,3-indanodiones **77** (Scheme 26). It has been found that the intermediate **79** underwent the Stetter reaction to form the enolate intermediate **80**, which next was transformed to the intermediate **81** via aldol condensation. The release of NHC gave the β-hydroxy ketone **82**, which was deprotonated to enolate **83**. The latter underwent a Michael cyclization reaction to afford **84**. Finally, dehydratation of **84** gave the spiro bis-indane product **85** (Scheme 27).

**Scheme 28:**
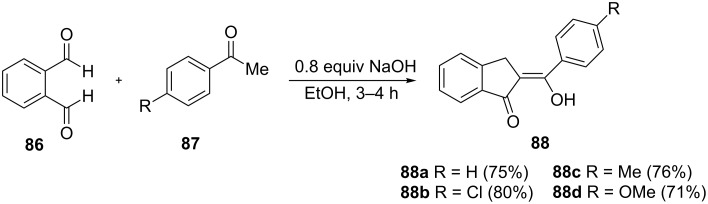
Synthesis of 2-benzylidene-1-indanone derivatives **88a**–**d**.

A new method to synthesize 2-benzylidene-1-indanone derivatives **88a**–**d** has been proposed in 2014 by Álvarez-Toledano et al. [52]. These derivatives were obtained from the reaction of *o*-phthalaldehyde (**86**) with acetophenone **87** (Scheme 28). Iron(III) complexes of **88a**–**d** turned out to be promising candidates for potential photovoltaic or luminescence applications.

**Scheme 29:**
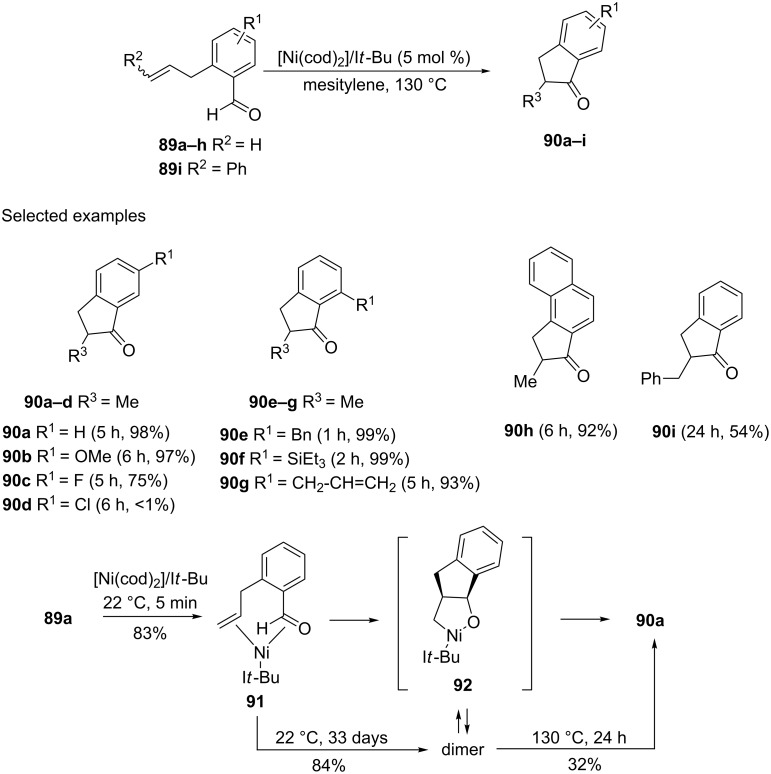
Synthesis of 1-indanone derivatives **90a–i**.

An intramolecular hydroacylation, catalyzed by nickel(0)/*N*-heterocyclic carbenes leading to the formation of a variety of 1-indanones and 1-tetralones has been proposed by Ogoshi et al. [53]. Thus, hydroacylation of *o*-allylbenzaldehyde derivatives **89** in the presence of [Ni(cod)_2_] and the *N*-heterocyclic carbene with an I*t-*Bu substituent gave 1-indanones **90a**–**i** in high yields (Scheme 29). In the case of **90**, it has been proved that this reaction proceeds with participation of Ni-complex **91** isolated in 83% yield which next was converted to 1-indanone **90a** via the monomeric complex **92** or its dimer.

**Scheme 30:**
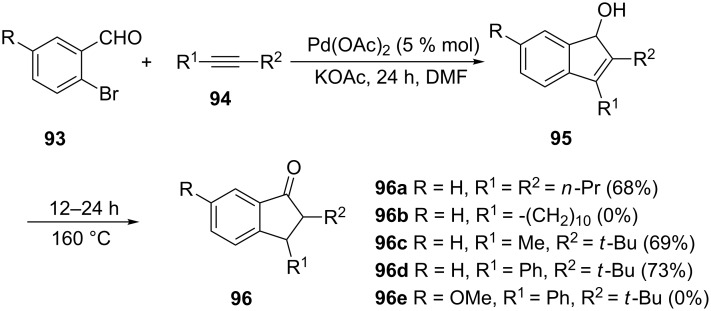
Synthesis of 1-indanones **96** from *o*-bromobenzaldehydes **93** and alkynes **94**.

*o*-Bromobenzaldehyde **93**, in the presence of a palladium catalyst, underwent intermolecular carbopalladation with alkynes **94**, followed by intramolecular nucleophilic vinylpalladation to give indenol derivatives **95** [54]. Further heating of **95** led to isomerization of the double bond to give the corresponding 1-indanones **96** (Scheme 30).

**Scheme 31:**
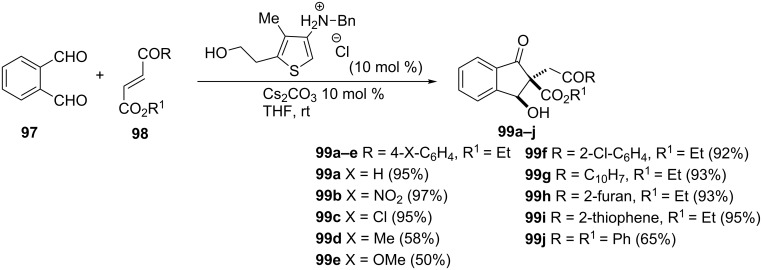
Synthesis of 3-hydroxy-1-indanones **99**.

3-Hydroxy-1-indanones **99a–j** have been applied in the synthesis of human papilloma virus type 11 (HPV11) inhibitors. These 1-indanones **99** have been synthesized using a *N*-heterocyclic carbene-catalyzed [4 + 1] annulation utilizing phthalaldehyde (**97**) and 1,2-diactivated Michael acceptors **98** (Scheme 31) [55–56].

**Scheme 32:**
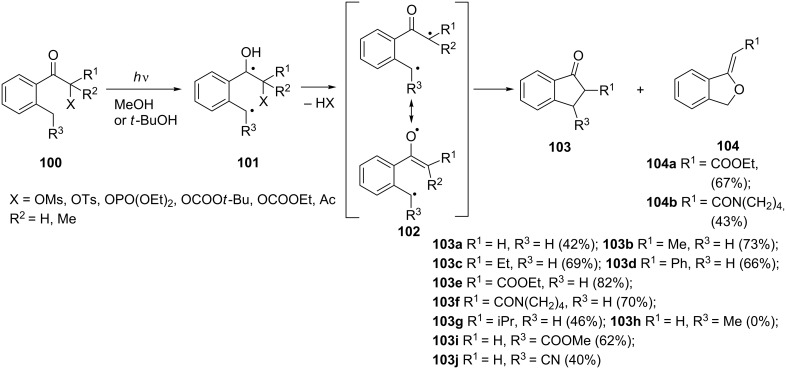
Photochemical preparation of 1-indanones **103** from ketones **100**.

**1.1.5 From ketones and 1,2-diketones:** Another interesting approach to 1-indanones **103** has been proposed by Wessig et al. [57]. The authors have used a photochemical cyclization of ketones **100** containing good leaving groups X adjacent to the carbonyl group. As a result of irradiation, 1,4-diradicals **101** have been formed from ketones **100** through n–π* excitation followed by 1,5-hydrogen migration involving the *o*-alkyl substituent. 1,5-Diradicals **102** were formed as a result of elimination of acid HX from 1,4-diradicals **101**. Finally, 1,5-diradicals **102** underwent cyclization to give 1-indanones **103** in good yields and 2-alkylidene benzo[c]furan derivatives **104** as byproducts (Scheme 32).

**Scheme 33:**
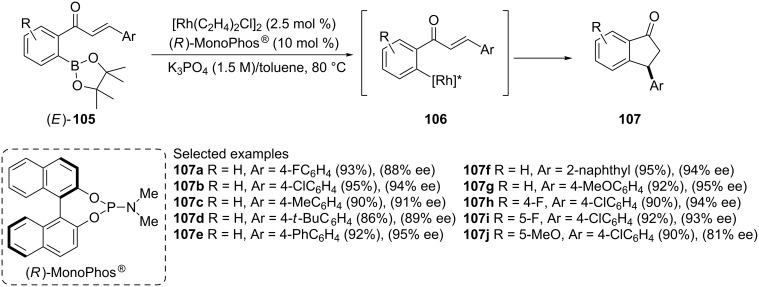
Synthesis of chiral 3-aryl-1-indanones **107**.

Chiral 3-aryl-1-indanones **107** have been synthesized via rhodium-catalyzed asymmetric cyclization of pinacolborane chalcone derivatives **105** using (*R*)-MonoPhos^®^ as a chiral ligand [58]. In this reaction, a wide variety of 1-indanones **107** were obtained in high yields and up to 95% enantiomeric excess (Scheme 33).

**Scheme 34:**
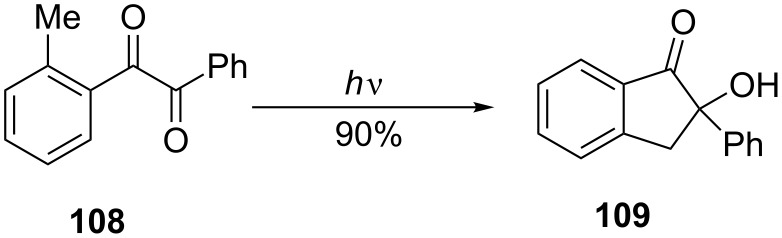
Photochemical isomerization of 2-methylbenzil **108**.

2-Methylbenzil (**108**) has been converted to 2-hydroxy-2-phenylindan-1-one (**109**) as a result of photochemical isomerization, in 90% yield (Scheme 34) [59].

**Scheme 35:**
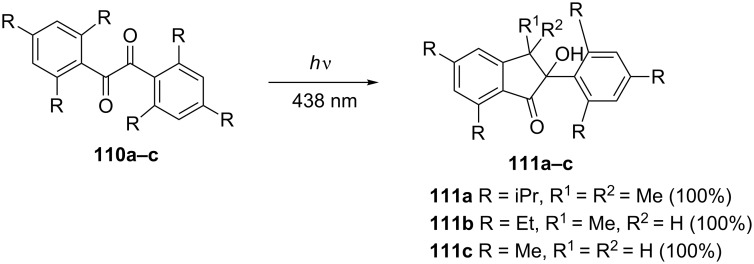
Synthesis of 2-hydroxy-1-indanones **111a–c**.

Wagner et al. have reported that hexaisopropyl-, hexaethyl- and hexamethylbenzils **110a–c** photocyclized to the corresponding 2-hydroxy-1-indanones **111a–c** (Scheme 35) [60].

**Scheme 36:**
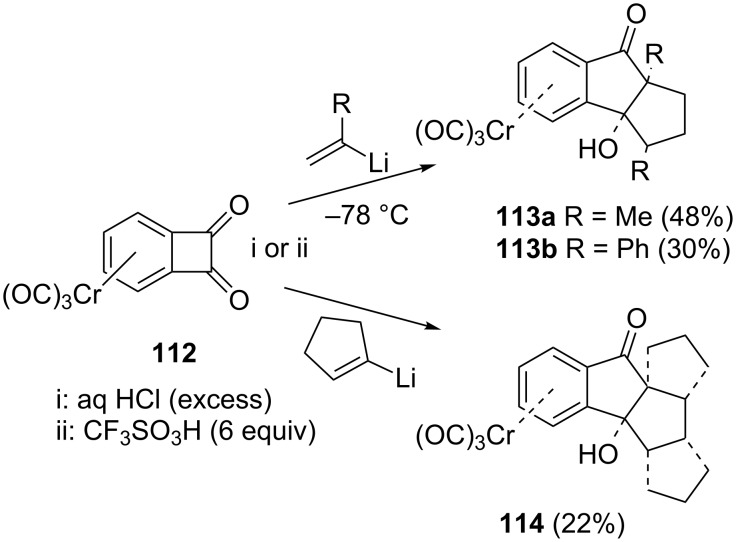
Synthesis of 1-indanone derivatives **113** and **114** from η^6^-1,2-dioxobenzocyclobutene complex **112**.

The chromium η^6^-1,2-dioxobenzocyclobutene complex **112** could be converted into 1-indanones **113** and **114** by addition of vinyllithium derivatives, followed by a double anionic oxy-Cope rearrangement under mild conditions (Scheme 36) [61]. The derivative **114** turned out to be particularly interesting because of its application in the synthesis of anticancer compounds [62].

**Scheme 37:**
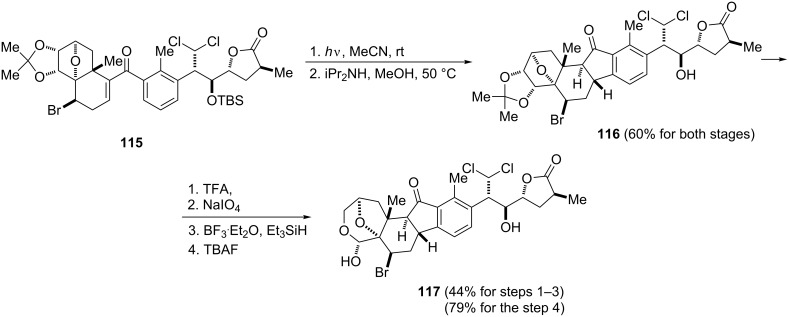
Synthesis of nakiterpiosin (**117**).

**1.1.6 From 1,3-dienones:** The major role in the synthesis of 1-indanones plays the Nazarov reaction of 1,3-dienones in which one of two double bonds is derived from the aromatic system. Nakiterpiosin (**117**) is a marine sponge metabolite which demonstrates a potent cytotoxicity against the P388 leukemic cell line. The photo-variant of the Nazarov cyclization has been applied as one of the steps in the total synthesis of nakiterpiosin (**117**, Scheme 37) [63]. Starting from substrate **115**, 1-indanone **116** was isolated in 60% yield and further used in the synthesis of the natural product.

**Scheme 38:**
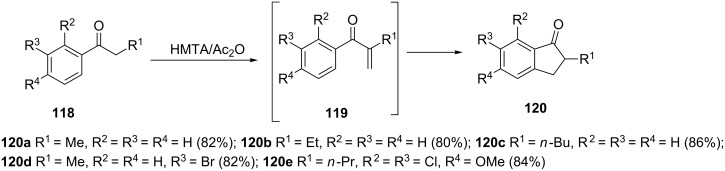
Synthesis of 2-alkyl-1-indanones **120**.

Hexamethylenetetramine (HMTA) is a commonly used promoter of aryl alkyl ketones in the Mannich reaction which has been applied in the synthesis of α,β-unsaturated ketones **119** [64]. The HMTA/acetic anhydride-promoted α-methylenation of compounds **118** followed by cyclization of the resulting enones **119** allowed to obtain a series of 2-alkyl-1-indanones **120** in very good yields (Scheme 38).

**Scheme 39:**
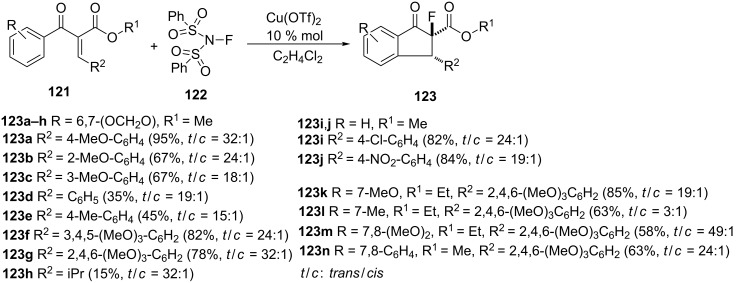
Synthesis of fluorine-containing 1-indanone derivatives **123**.

A stereoselective, catalytic, tandem transformation of α,β-unsaturated arylketones **121** to fluorine-containing 1-indanone derivatives **123** via the Nazarov cyclization followed by electrophilic fluorination, has been described in 2007 by Ma et al. [65]. This reaction was catalyzed by Cu(II) triflate and proceeded in the presence of *N*-fluorobenzenesulfonimide (NFSI) **122** as a fluorinating reagent (Scheme 39).

**Scheme 40:**
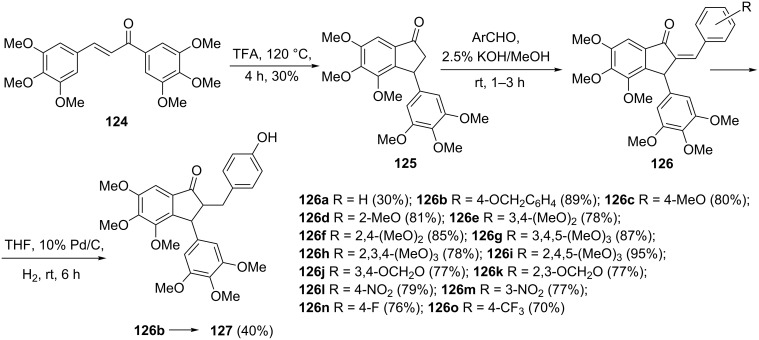
Synthesis of 2-benzylidene and 2-benzyl-1-indanones **126**, **127** from the chalcone **124**.

Scientists are tirelessly exploring for better anticancer pharmaceuticals. Negi et al. have joined these efforts and proposed a synthesis of 2-benzylidene-1-indanones, which exhibited a strong cytotoxicity against four human cancer cell lines: breast (MCF-7), colon (HCT), leukemia (THP-1) and lung (A549) with IC_50_ values in the range of 10–880 nM [66]. The synthesized compounds have also shown a strong inhibition of tubulin polymerase with IC_50_ values in the range of 0.62–2.04 µM. In this synthesis, the chalcone **124** underwent a Nazarov cyclization in the presence of trifluoroacetic acid to give 1-indanone **125**. 2-Benzylidene-1-indanones **126a–o** were obtained by a Knoevenagel condensation of 1-indanone **125** with various aromatic aldehydes. Hydrogenolysis of 2-benzylidene-1-indanone **126b** using Pd/C allowed to obtain 2-benzyl substituted 1-indanone **127** (Scheme 40).

**Scheme 41:**
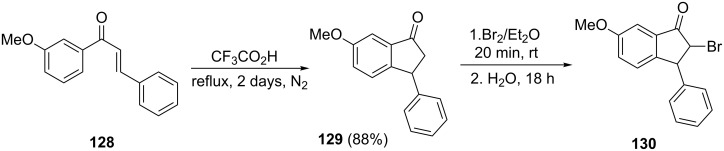
Synthesis of 2-bromo-6-methoxy-3-phenyl-1-indanone (**130**).

2-Bromo-6-methoxy-3-phenyl-1-indanone **130**, as an interesting bromo reagent for further transformations, has been synthesized from chalcone **128** [67]. In this synthesis, a Nazarov reaction of chalcone **128**, in the presence of trifluoroacetic acid, gave 6-methoxy-3-phenyl-1-indanone **129** in 88% yield followed by the reaction with bromine in diethyl ether to give 2-bromo-6-methoxy-3-phenyl-1-indanone (**130**, Scheme 41).

**Scheme 42:**
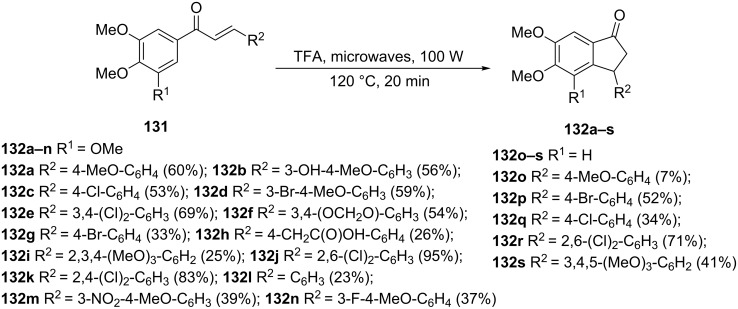
Synthesis of combretastatin A-4-like indanones **132a–s**.

An efficient microwave-assisted synthesis of 1-indanones **132a–s** related to combretastatin A-4 has been proposed by Lawrence et al. [68]. Two of the indanones were obtained via a Nazarov cyclization of chalcones **131** without using microwaves, in the presence of trifluoroacetic acid (TFA) at 120 °C (4 hours). The authors have proved that the microwave heating would significantly shorten the reaction time up to 20 minutes under the same reaction conditions (TFA, 120 °C, Scheme 42). The cell growth inhibitory properties of the synthesized 1-indanones **132a–s** were also investigated on the K562 human chronic myelogenous leukaemia cell line. The strongest cytotoxicity against the K562 cell line showed the following compounds: **132a**, **132b**, **132d**, **132f, 132g**, **132i**, **132k**, **132m**, **132n**, **132o** with IC_50_ values in the range of 0.08–2.8 µM. The compound **132b** demonstrated the greatest resemblance to combretastatin A-4.

**Figure 3:**
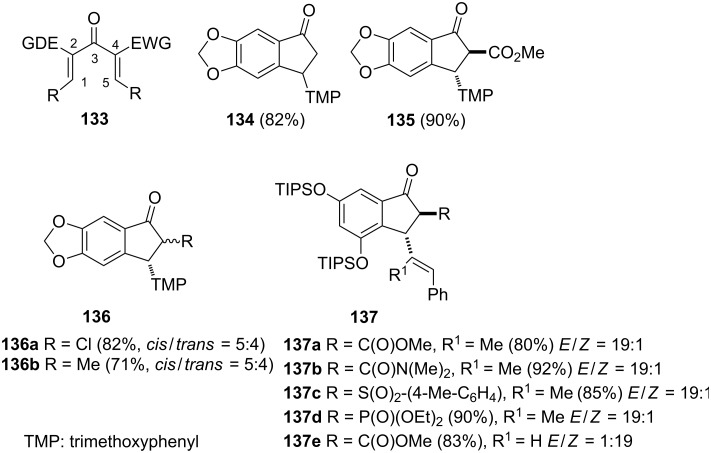
Chemical structures of investigated dienones **133** and synthesized cyclic products **134–137**.

In 2008, Frontier et al. have examinated the impact of the dienone substitution in Nazarov cyclizations [69]. They synthesized a series of the Nazarov substrates **133** with electron-donating substituents at C-2 and electron-withdrawing substituents at C-4. By using catalytic amounts of Cu(OTf)_2_ or Cu(ClO_4_)_2_ as Lewis acids, cyclic products **134**–**137** have been obtained as single diastereoisomers in high yields (Figure 3). It has been proven that the reactivity and the selectivity of this cyclization can be controlled by positioning of the dienone **133** substituents. In the previous studies of the reductive Nazarov cyclization, similar results were obtained – two *E* and *Z* dienone isomers were converted into one diastereoisomeric product [70–71].

**Figure 4:**
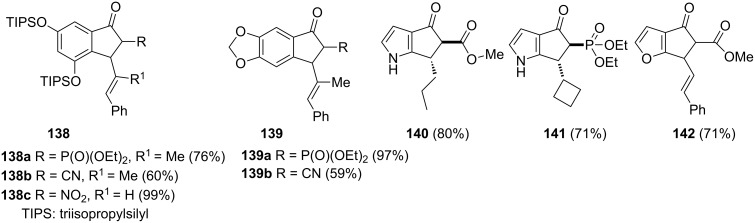
Chemical structures of 1-indanones and their heteroatom analogues **138**–**142**.

A dicationic iridium(III)-catalyzed Nazarov cyclization has been applied for the synthesis of functionalized 1-indanones and their heteroatom analogues **138–142** which may be further converted into biologically active compounds (Figure 4) [72]. Products **138–142** were obtained by electrocyclization of the substrates substituted by electron-withdrawing groups, such as CO_2_Me, P(O)(OEt)_2_, CN or NO_2_. This reaction was carried out in the presence of an iridium catalyst and antimony hexafluoride (AgSbF_6_) under mild conditions. The starting chalcones were almost completely converted into 1-indanones **138–142** and isolated in very good yields.

**Scheme 43:**
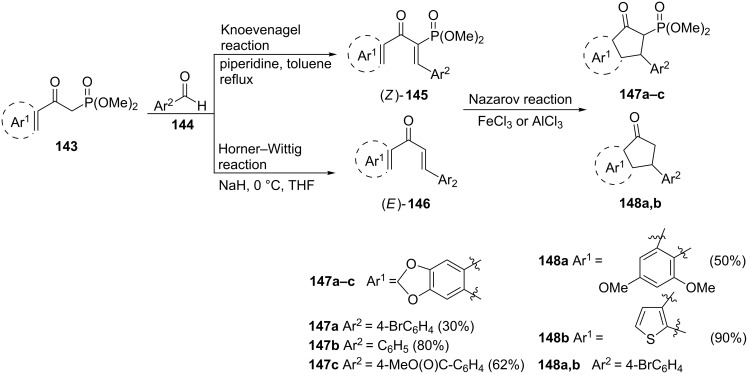
Synthesis of 2-phosphorylated and 2-non-phosphorylated 1-indanones **147** and **148** from β-ketophosphonates **143**.

Our research group synthesized 3-aryl-1-indanones **148** and previously unknown 3-aryl-2-phosphoryl-1-indanones **147** which exhibited anticancer activity against HeLa and K562 cell lines at the µM level [73]. Both groups of products have been obtained from the corresponding phosphorylated chalcones (*Z*)-**145** or nonphosphorylated chalcones (*E*)-**146**, in selective Horner–Wittig or Knoevenagel olefinations, followed by a Nazarov cyclization using FeCl_3_ or AlCl_3_. The 2-phosphorylated chalcones (*Z*)-**145** and non-phosphorylated ones (*E*)-**146** could be obtained from the same substrates, β-ketophosphonates **143** and aromatic aldehydes **144** depending on the reaction conditions used (piperidine/toluene for the Knoevenagel reaction or NaH/THF for the Horner–Witting reaction) (Scheme 43).

**Scheme 44:**
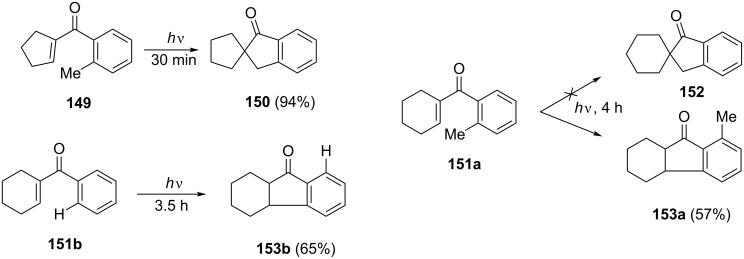
Photochemical synthesis of 1-indanone derivatives **150**, **153a**, **153b**.

Photochemical reactions play an important role in the synthesis of 1-indanone derivatives. Thus, photolysis of the ketone **149** gave the 1-indanone **150** in 94% yield. It is worth mentioning that photolysis of the ketone **151a** did not lead to the formation of 1-indanone **152** corresponding to **150** but led to the derivative **153a** (Scheme 44) [74].

**Scheme 45:**
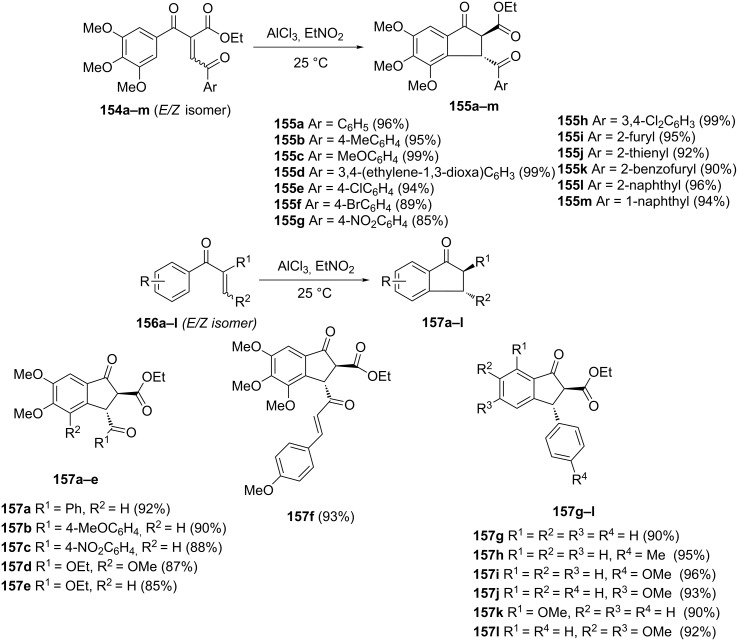
Synthesis of polysubstituted-1-indanones **155**, **157**.

The Nazarov-type cyclization has been proposed for the synthesis of polysubstituted-1-indanones **155a–m**, and **157a–l** [75]. They were obtained from 1,4-enediones **154** and aryl vinyl β-ketoesters **156** in the presence of AlCl_3_ as a promoter, in high yields (up to 99%) (Scheme 45). It was further proved that the pattern of substituents at C-2, C-4 and C-5 positions was essential for the reaction efficiency.

#### 1.2 From alcohols

**Scheme 46:**
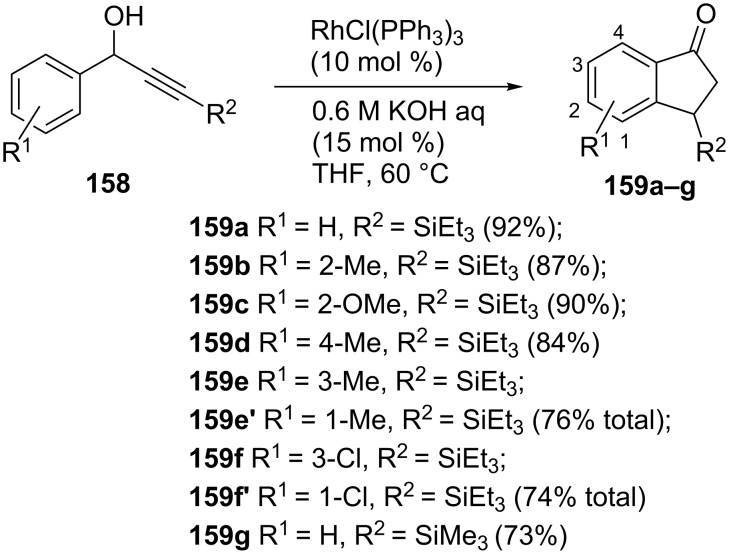
Synthesis of 1-indanones **159a–g** from α-arylpropargyl alcohols **158** using RhCl(PPh_3_)_3_ as a catalyst.

An interesting synthesis of optically active 1-indanones **159a–g** by a rhodium-catalyzed isomerization of racemic α-arylpropargyl alcohols **158** has been developed by Shintani, Okamoto and Hayashi (Scheme 46) [76]. By the mechanistic investigations using deuterium-labeled substrates, the authors have disclosed that the methine proton of the alcohol goes to the β-position of the 1-indanone, while the *ortho*-proton of the phenyl group is shifted to the α-position.

**Scheme 47:**
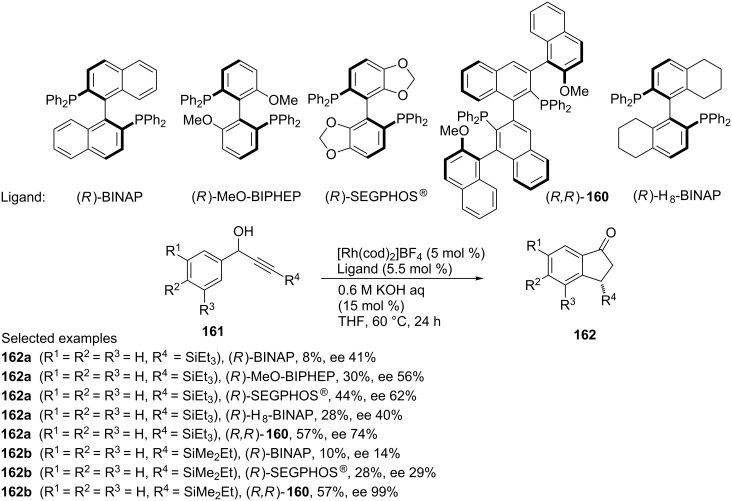
Synthesis of optically active 1-indanones **162** via the asymmetric Rh-catalyzed isomerization of racemic alcohols **161** using optically pure bisphosphine ligands and Rh(cod)_2_BF_4_ as a catalyst.

**Scheme 48:**
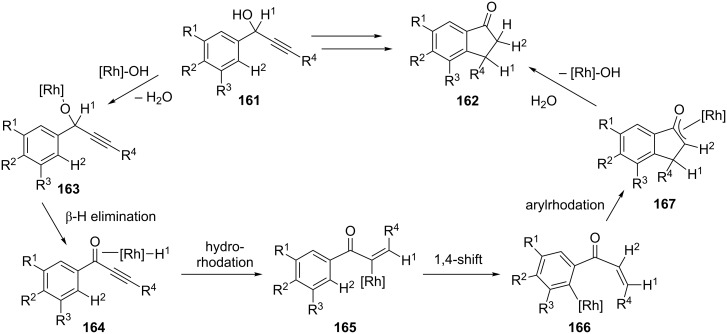
Mechanism of the Rh-catalyzed isomerization of α-arylpropargyl alcohols **161** to 1-indanones **162**.

The same research group has proposed another asymmetric isomerization of racemic alcohols **161** leading to the formation of 1-indanones **162** [77]. In this reaction, β-chiral 1-indanones **162** were obtained by isomerization of racemic α-arylpropargyl alcohols **161** in the presence of a rhodium catalyst. A high enantioselectivity has been achieved by the use of the chiral bisphosphine ligand (*R,R*)-**160** (Scheme 47). A catalytic cycle of this isomerization is shown in Scheme 48. First, alkoxorhodium **163**, next alkenylrhodium **165** were formed as intermediates as a result of dehydration and β-H elimination followed by hydrorhodation, respectively. Then, rhodium 1,4-migration, alkylrhodation and finally rhodium elimination led to 1-indanones **162** via intermediates **166** and **167**.

**Figure 5:**
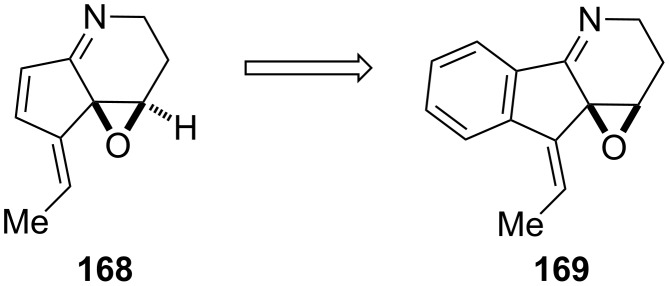
Chemical structure of abicoviromycin (**168**) and its new benzo derivative **169**.

**Scheme 49:**
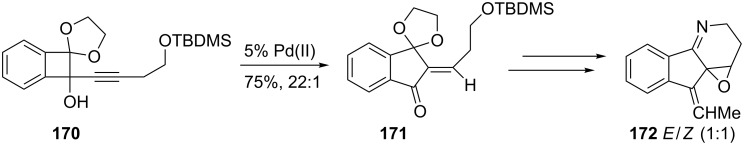
Synthesis of racemic benzoabicoviromycin **172**.

Abicoviromycin (**168**) is an antiviral and antifungal molecule produced by bacteria. Because of its interesting biological activity, Mitchell and Liebeskind have decided to synthesize abicoviromycin (**168**) derivatives [78]. In spite of the potent biological activity, abicoviromycin (**168**) is extremely heat- and acid-sensitive. Moreover, this compound polymerizes rapidly even at low temperatures, such as −50 °C. Therefore, until 1989 abicoviromycin (**168**) has not been successfully synthesized. The unit which probably determines the reactivity and unstability of abicoviromycin (**168**) is the diene-imine fragment. Due this fact, the authors have decided to replace the double bond at the 6,7 position by the benzene ring in the new abicoviromycin derivative **169** to increase the stability while still retaining the biological activity (Figure 5). Thus, the palladium-catalyzed ring expansion of 2-alkynyl-2-hydroxybenzocyclobutenone **170** allowed to obtain alkylidenoindanedione intermediate **171**, which was further converted into racemic benzoabicoviromycin **172** (Scheme 49). The racemic benzoabicoviromycin **172** as well as its (*Z*)-ethylidene stereoisomer have been screened for in vitro biological activity (antiviral, anticancer and antifungal). The significant in vitro cytotoxicity was observed against the following cell lines: A549 (IC_50_: 5.48–5.01 mg/mL), A549/VP (IC_50_: 4.76–4.18 mg/mL), B16-PRIM (IC_50_: 0.16 mg/mL), HCT116 (IC_50_: 1.47–141 mg/mL), HCT/VP35 (IC_50_: 1.26–1.16 mg/mL). Unfortunately, these levels of activity turned out to be useless in vivo. However, considering the enormous potential of abicoviromycin, syntheses of its additional analogs are reasonable.

#### 1.3 From alkyl chlorides

**Scheme 50:**
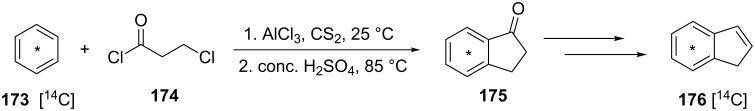
Synthesis of [^14^C]indene **176**.

Radiolabeled tracers can supply precious information about structures of biocatalytic reaction networks. The [^14^C]1-indanone **175** has been used in the synthesis of the [^14^C]indene **176**, which was next applied for the examination of the indene bioconversion network expressed in *Rhodococcus* sp. KY1 [79]. The [^14^C]1-indanone **175** was obtained in the one-pot synthesis involving the Friedel–Crafts acylation of [^14^C]benzene **173** with chloropropionic acid chloride **174** followed by a Friedel–Crafts cyclization in the presence of concentrated H_2_SO_4_. The [^14^C]1-indanone **175** was then converted in three steps to [^14^C]indene **176** (Scheme 50).

**Scheme 51:**
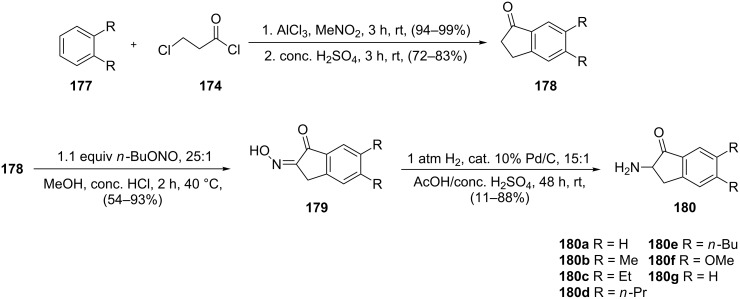
Synthesis of indanone derivatives **178**–**180**.

The same reaction sequence involving the Friedel–Crafts acylation of disubstituted benzene derivatives **177** with 3-chloropropionyl chloride **174** followed by a intramolecular Friedel–Crafts alkylation afforded 1-indanones **178** (Scheme 51) [80]. A direct reaction of the latter with *n*-butylnitrite led to the formation of keto-oximes **179** which underwent a Pd/C catalytic reduction to give 2-amino substituted 1-indanones **180**. Both keto-oximes **179** and 2-amino derivatives **180** are β_2_-adrenergic agonists tested for bronchodilating activity.

**Scheme 52:**
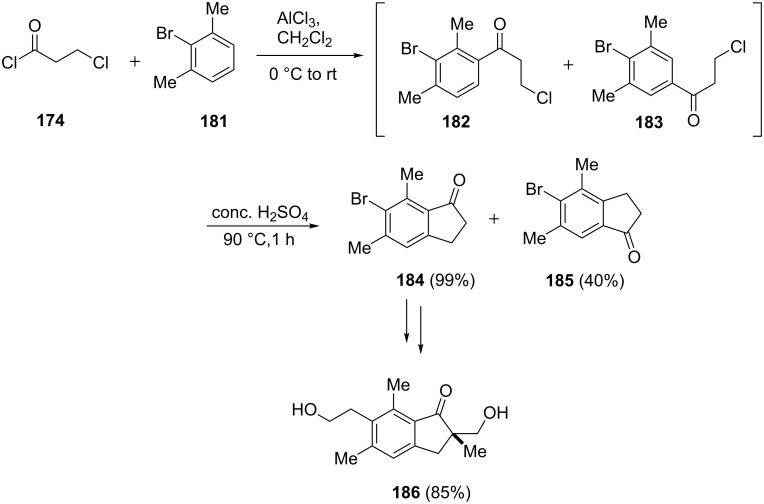
Synthesis of racemic pterosin A **186**.

The pterosin family are sesquiterpenoids naturally occurring in bracken fern (*Pteridium aquilinum*), some of them exhibit antibacterial and cytotoxic activity. A practical synthesis of pterosin A (**186**), being a 1-indanone derivative, has been proposed by Uang et al. [81]. In this synthesis, 3-chloropropionyl chloride (**174**) reacted with 2-bromo-1,3-dimethylbenzene (**181**) in the presence of AlCl_3_ to give two isomeric products **182** and **183**. The mixture of **182** and **183** was heated with concentrated H_2_SO_4_ at 90 °C to form the corresponding 1-indanones **184** and **185** (in 39% and 40% yield, respectively). The 1-indanone **184** was converted to pterosin A (**186**) in a sequence of reactions (Scheme 52).

#### 1.4 From alkynes

**Scheme 53:**
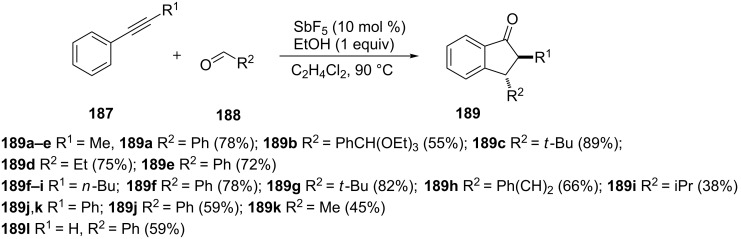
Synthesis of *trans*-2,3-disubstituted 1-indanones **189**.

The use of a catalytic amount of antimony pentafluoride and ethanol converted mixtures of phenylalkynes **187** and aldehydes **188** to *trans*-2,3-disubstituted 1-indanones **189** in the one-pot reaction (Scheme 53) [82].

**Scheme 54:**
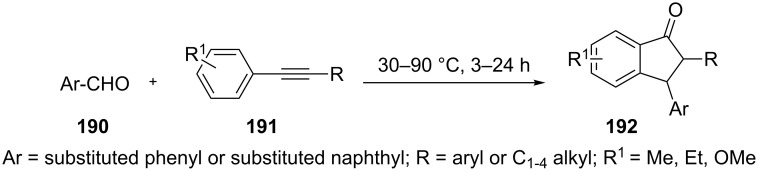
Synthesis of 3-aryl-1-indanone derivatives **192**.

A synthesis of 3-aryl-1-indanone derivatives **192** from aromatic aldehydes **190** and alkyne derivatives **191** has been patented by Xi and Liu in 2016 [83]. The reaction proceeded in the presence of methyl trifluoromethanesulfonate, in dichloromethane or 1,2-dichloroethane in high yields (Scheme 54).

#### 1.5 From nitriles

**Scheme 55:**
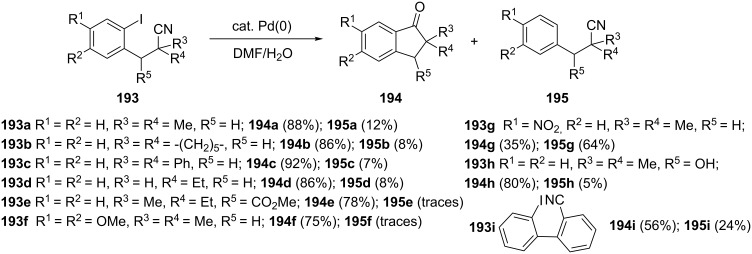
Synthesis of 1-indanone derivatives **194** from 3-(2-iodoaryl)propanonitriles **193**.

Nitrile derivatives are also useful substrates for the synthesis of 1-indanones. An efficient synthesis of 1-indanones **194** via palladium-catalyzed cyclization of 3-(2-iodoaryl)propanenitriles **193** has been described by Pletnev and Larock [84]. This reaction was compatible with a wide variety of electron-donor and electron-acceptor functional groups. The authors have also found that the formation of 1-indanones **194** is accompanied by the reduction of the carbon–iodine bond in 3-(2-iodoaryl)propanonitriles **193** leading to the formation of the nitrile **195** as a byproduct (Scheme 55).

**Scheme 56:**
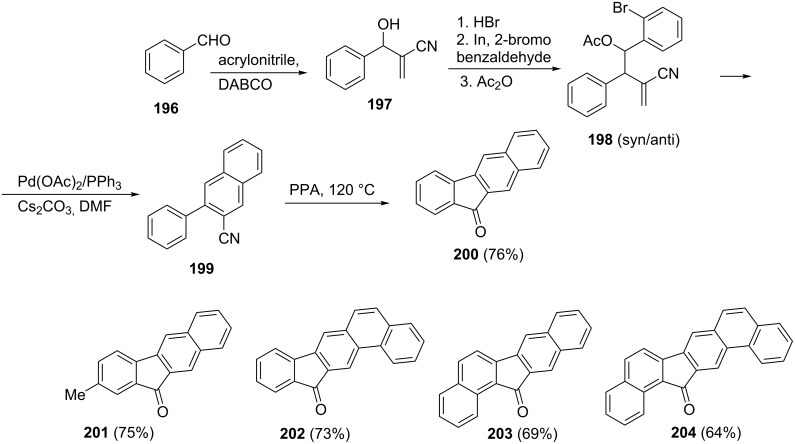
Synthesis of 1-indanones **200**–**204** by cyclization of aromatic nitriles.

Cyclization of 3-phenylnaphtalene-2-carbonitrile (**199**) in the presence of polyphosphoric acid gave 1-indanone **200** in 76% yield. The nitrile **199** was obtained from benzaldehyde **196** as a result of sequencial reactions leading to intermediates **197** and **198** [85]. The authors have also synthesized other fluorenone derivatives **200**–**204** by using this method (Scheme 56).

#### 1.6 From diazo compounds

**Scheme 57:**
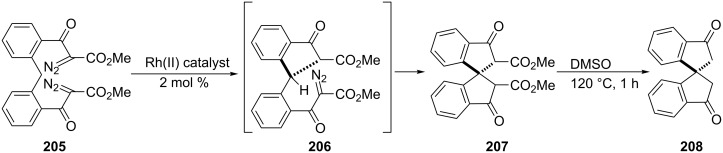
Synthesis of 1,1’-spirobi[indan-3,3’-dione] derivative **208**.

Hashimoto et al. have proposed the synthesis of optically active 1,1’-spirobi[indan-3,3’-dione] derivative **208** (up to 80% enantiomeric excess) from bis(α-diazo-β-keto ester) **205** [86]. The key step of this synthesis was a double intramolecular C–H insertion process catalyzed by dirhodium(II) tetrakis[*N*-phthaloyl-(*R* or *S*)-*tert*-leucinate]. The resulting spiroindanone derivative **207** obtained from the intermediate **206**, underwent demethoxycarbonylation to give 1,1’-spirobi[indan-3,3’-dione] **208** (Scheme 57).

**Scheme 58:**
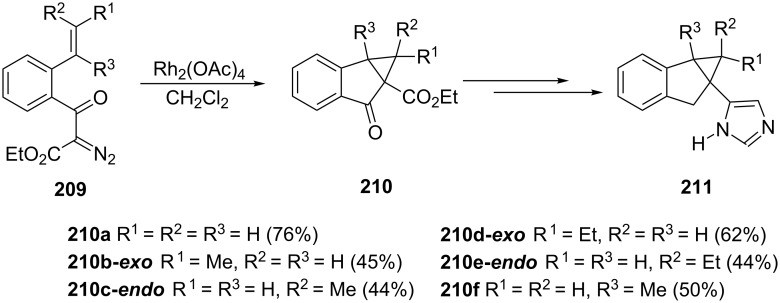
Total synthesis of atipamezole analogues **211**.

Atipamezole is a synthetic α_2_-adrenergic receptor antagonist used in veterinary for reversal of the sedative and analgesic effects induced by α_2_-adrenergic receptor agonists. Vacher et al. synthesized α_2_-adrenergic receptor antagonists, potentially more selective than known compounds [87]. Transformation of diazo compounds **209** into 1-indanone derivatives **210**, catalyzed by rhodium acetate, has been one of the steps in the total synthesis of atipamezole analogues **211** (Scheme 58).

**Scheme 59:**
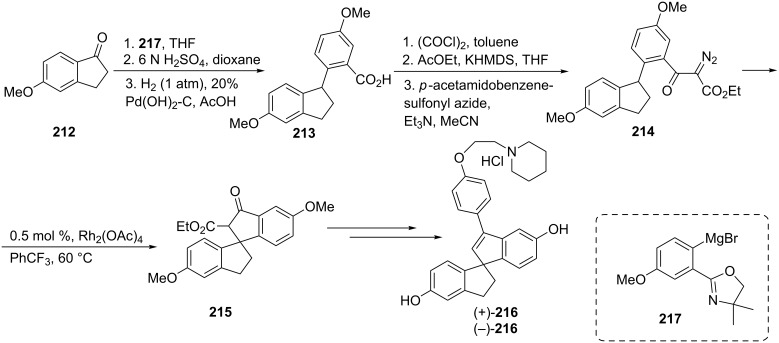
Synthesis of 3-[4-(1-piperidinoethoxy)phenyl]spiro[indene-1,1’-indan]-5,5’-diol hydrochloride **216**.

The most common symptom of the menopause is hot flash, which is characterized by sweating, sudden feeling of heat, palpitation or anxiety. Hormone replacement therapy (HRT) alleviates above mentioned symptoms but its use has been limited because of many side effects, such as increased hormone-dependent cancers risk. Watanabe et al. have synthesized a selective estrogen receptor modulator, 3-[4-(1-piperidinoethoxy)phenyl]spiro[indene-1,1’-indane]-5,5’-diol hydrochloride (**216**) which may be used for a new treatment of hot flush [88]. In this synthesis, the reaction of 5-methoxyindan-1-one (**212**) with the Grignard reagent **217** followed by acid-catalyzed dehydration and hydrogenolysis of the resulting double bond with Pd(OH)_2_/C, gave benzoic acid **213**. Next, the latter was converted to α-diazo-β-keto ester **214** which then was submitted to the rhodium(II) acetate catalyzed intramolecular, carbon–hydrogen insertion reaction to give the spiroindane **215**. Finally, the spiroindane **215** was converted to the expected, estrogen receptor modulator **216** (Scheme 59).

**Scheme 60:**
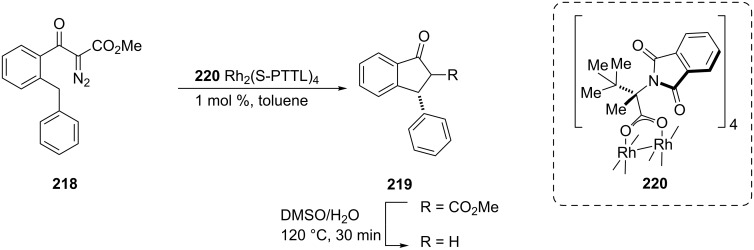
Synthesis of 3-arylindan-1-ones **219**.

3-Arylindan-1-ones **219**, versatile intermediates for the synthesis of a number of biologically active compounds, have been synthesized from α-diazo-β-keto ester **218** via a intramolecular C–H insertion reaction catalyzed by the rhodium(II) complex **220** followed by the carboxylic methyl ester hydrolysis/decarboxylation in DMSO/H_2_O at 120 °C with up to 72% enantiomeric excess (Scheme 60) [89].

#### 1.7 From epoxides and cyclopropanes

**Scheme 61:**
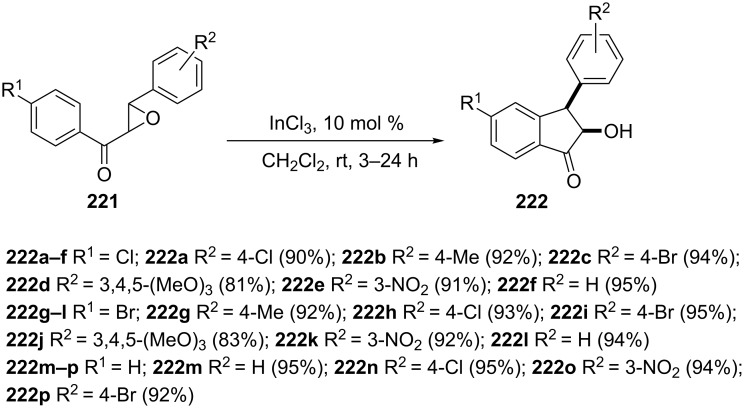
Synthesis of 2-hydroxy-1-indanones **222**.

The chalcone epoxides **221** ring opening catalyzed by indium(III) chloride, followed by a intramolecular Friedel–Crafts alkylation has been used by Ahmed et al. for the synthesis of 2-hydroxyindan-1-one derivatives **222** in good yields (Scheme 61) [90].

**Scheme 62:**
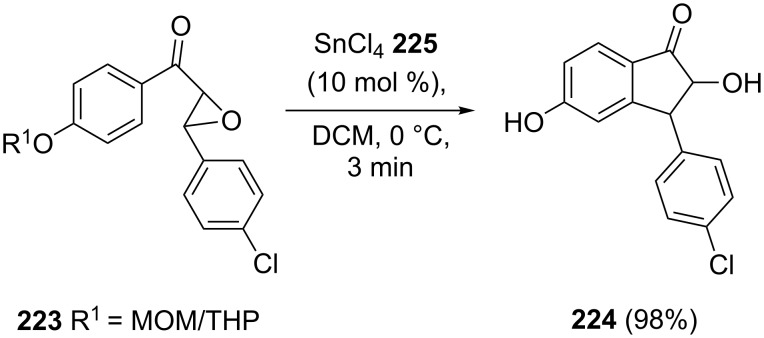
Synthesis of the 1-indanone **224** from the THP/MOM protected chalcone epoxide **223**.

The same research group used the THP (tetrahydropyranyl) and MOM (methoxymethyl) protected chalcone epoxides and tin(IV) chloride under mild conditions to synthesize dihydroxy substituted 1-indanones [91]. All reactions have been completed within 2–3 min at 0 °C in the presence of the catalyst Sn(IV)Cl_4_ (**225**) and gave products in excellent yields (90–98%). For example, 1-indanone derivative **224** has been obtained from the THP/MOM protected chalcone epoxide **223** in 98% yield for both THP and MOM ethers (Scheme 62).

**Scheme 63:**
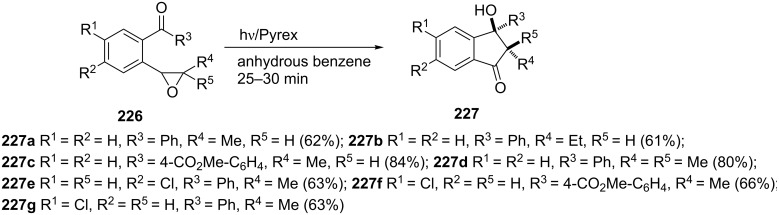
Synthesis of 1-indanones **227** from γ,δ-epoxy ketones **226**.

Irradiation of aromatic γ,δ-epoxy ketones **226** with a medium-pressure UV mercury lamp (450 W) led to the formation of 1-indanones **227** via a photochemical epoxy rearrangement and 1,5-biradical cyclization tandem reaction (Scheme 63) [92]. The best yields (up to 84%) were achieved by using substrates **226** with electron-acceptor substituents at the *para* position of the aryl group.

**Scheme 64:**
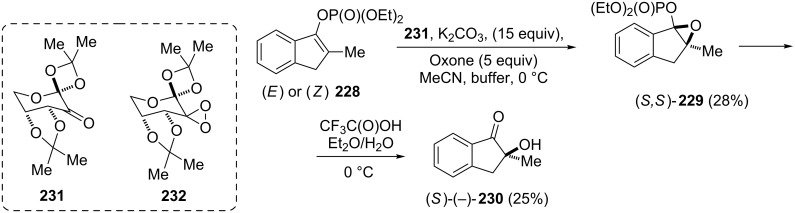
Synthesis of 2-hydroxy-2-methylindanone (**230**).

A new method for the synthesis of optically active α-hydroxy ketones by asymmetric oxidation of the enol phosphates catalyzed by Sharpless reagents or chiral dioxirane has been proposed by Krawczyk et al. [93]. For example, optically active 1-indanone **230** was obtained from the cyclic enol phosphate **228** which next was reacted with a fructose-derived dioxirane **232** generated in situ from the ketone **231**, to provide the epoxide **229** (Scheme 64). Then, the latter was hydrolyzed with CF_3_C(O)OH in Et_2_O/H_2_O at 0 °C to obtain optically active 1-indanone **230**.

**Scheme 65:**
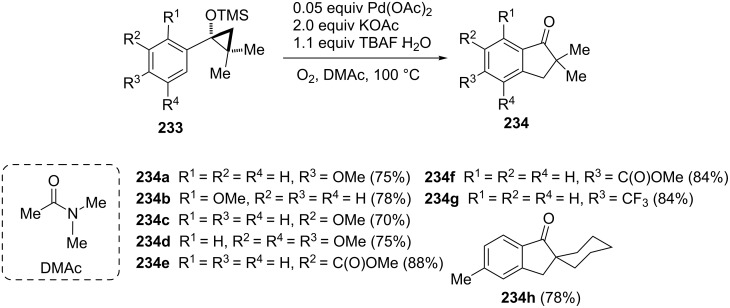
Synthesis of 1-indanone derivatives **234** from cyclopropanol derivatives **233**.

A very interesting approach for the synthesis of 1-indanones **234** based on the rearrangement of cyclopropanol derivatives **233**, has been reported in 2012 by Rosa and Orellana [94]. This reaction was carried out in the presence of palladium catalyst and gaseous oxygen as the terminal oxidant (Scheme 65).

#### 1.8 From other compounds

**Scheme 66:**
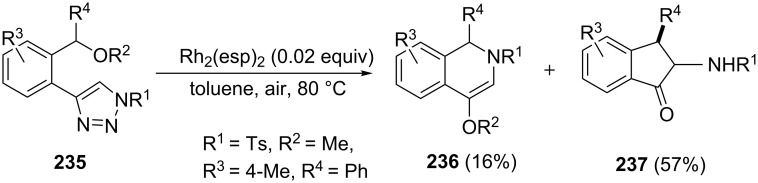
Synthesis of substituted 1-indanone derivatives **237**.

In 2016, Shi et al. have developed an unique, conditions-controlled [Rh_2_(esp)_2_] (esp = α,α,α’,α’-tetramethyl-1,3-benzenedipropionic acid)-catalyzed reaction of *N*-sulfonyl-1,2,3-triazoles **235** leading to a mixture of 1,2-dihydroisoquinolines **236** and substituted 1-indanone derivatives **237** via alkoxy group migration in 16 and 57% yields, respectively (Scheme 66) [95].

### 2 Construction of the 6-membered ring

The titles of subsections in this chapter contain names of the 1-indanone precursors which provide the biggest number of carbon atoms during the synthesis of the 1-indanone benzene ring. For instance, 1,3-dienes in the Diels–Alder reaction provide 4 carbon atoms of the six ones needed to construct the benzene ring of 1-indanone compared to dienophiles which deliver only two of them.

#### 2.1 From 1,3-dienes

**Scheme 67:**
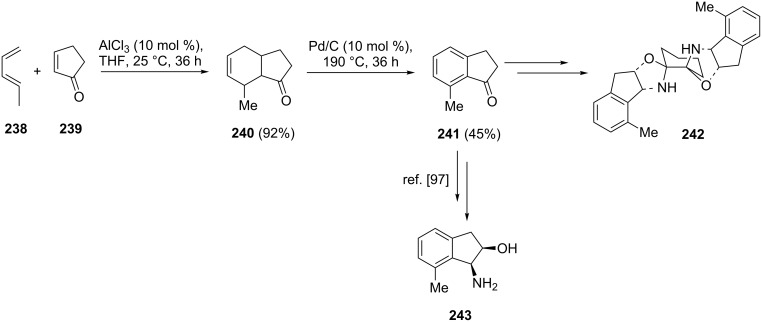
Synthesis of 7-methyl substituted 1-indanone **241** from 1,3-pentadiene (**238**) and 2-cyclopentenone (**239**).

Wolf and Xu have synthesized 7-methyl substituted 1-indanone **241** utilizing 1,3-pentadiene (**238**) and 2-cyclopentenone (**239**) as starting compounds [96]. 7-Methyl substituted 1-indanone **241** has been obtained in the Diels–Alder reaction between 1,3-pentadiene (**238**) and 2-cyclopentenone (**239**) followed by the oxidative aromatization with Pd/C (Scheme 67). The latter was further used as a substrate for the synthesis of bisoxazolidine ligand **242**. The same Diels–Alder reaction to obtain **241** has been used by Katsumura et al. [97]. In this case, **241** was further converted to *cis*-1-amino-7-methyl-2-indanol (**243**, Scheme 67).

**Scheme 68:**
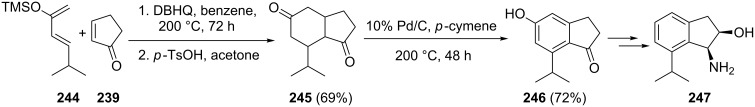
Synthesis of disubstituted 1-indanone **246** from the siloxydiene **244** and 2-cyclopentenone **239**.

Katsumura et al. have also synthesized disubstituted 1-indanone **246** using the Diels–Alder reaction [97]. This synthesis utilized the siloxydiene **244** and 2-cyclopentenone (**239**) which were reacted in the presence of 2,5-di-*tert*-butylhydroquinone (DBHQ) in benzene, followed by treatment with *p*-toluenesulfonic acid in acetone to give the diketone **245** (Scheme 68). Then, the latter underwent oxidative aromatization by treatment with Pd/C in *p*-cymene. The synthesized 1-indanone **246** was further converted to the *cis*-1-amino-2-indanol **247** and used as ligand for asymmetric reactions.

**Scheme 69:**
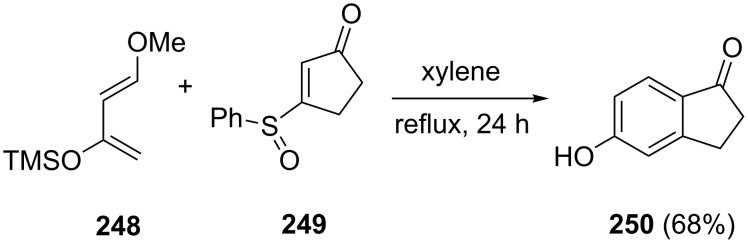
Synthesis of 5-hydroxy-1-indanone (**250**) via the Diels–Alder reaction of 1,3-diene **248** with sulfoxide **249**.

A similar way to synthesize 5-hydroxy substituted 1-indanone **250** by utilizing the 1,3-diene **248** and the sulfoxide **249**, has been described by Danishefsky et al. [98]. As a result of the cycloaddition, 5-hydroxy-1-indanone (**250**) has been obtained in 68% yield (Scheme 69).

**Scheme 70:**
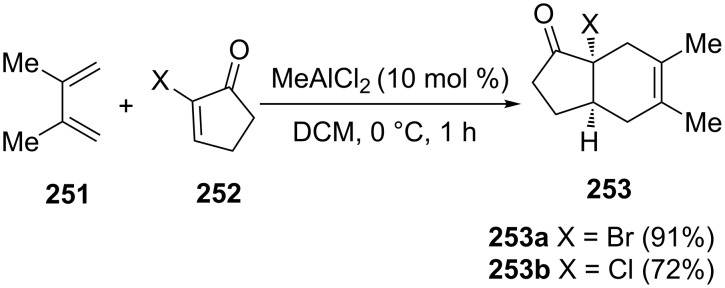
Synthesis of halogenated 1-indanones **253a** and **253b**.

Lee, Kim and Danishefsky have synthesized halogenated 1-indanones **253** from 2-halogenocyclopent-2-enones **252** and diene **251** [99]. As a result of the Diels–Alder reaction, bromo- and chloro-substituted 1-indanones **253a** and **253b** have been obtained in 91% and 72% yield, respectively (Scheme 70).

**Scheme 71:**
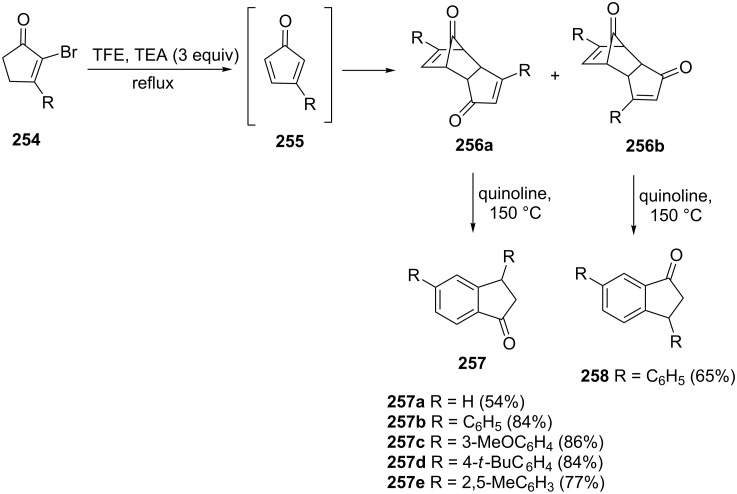
Synthesis of 1-indanones **257** and **258** from 2-bromocyclopentenones **254**.

Harmata et al. have synthesized 1-indanones **257** and **258** by utilizing 2-bromocyclopentenones **254** as starting materials [100]. First, the cyclopentadienone dimers **256a** and **256b** were generated from 2-bromocyclopentenones **254** using triethylamine (TEA) in trifluoroethanol (TFE). Then, the 1-indanones **257** and **258** were obtained from dimers **256a** or **256b** by heating in quinoline (Scheme 71).

**Scheme 72:**
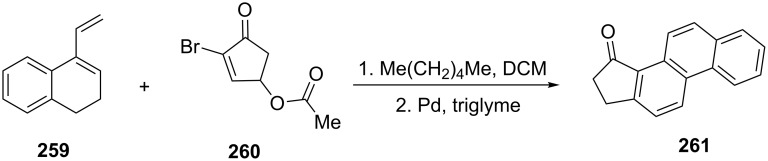
Synthesis of 1-indanone **261** from 2-bromo-4-acetoxy-2-cyclopenten-1-one (**260**) and 1,2-dihydro-4-vinylnaphthalene **259**.

Gacs-Baitz et al. have applied the Diels–Alder reaction between 1,2-dihydro-4-vinylnaphthalene (**259**) and 2-bromo-4-acetoxy-2-cyclopenten-1-one (**260**) to synthesize 1-indanone derivative **261** (Scheme 72) [101].

**Scheme 73:**
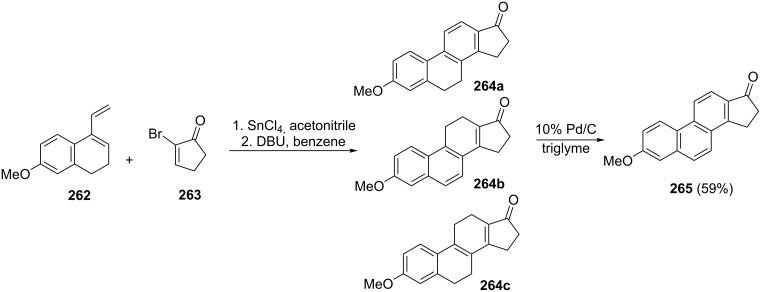
Synthesis of 1-indanone **265** from 1,2-dihydro-7-methoxy-4-vinylnaphthalene (**262**) and bromo-substituted cyclopentenone **263**.

Koreeda and Woski have synthesized the cyclopenta[α]phenanthrene derivative **265** having the steroid framework from 1,2-dihydro-7-methoxy-4-vinylnaphthalene (**262**) and α-bromo substituted cyclopentenone **263** by the SnCl_4_-catalyzed Diels–Alder cycloaddition [102]. In this reaction, 1-indanone **265** was obtained in 59% yield via dehydrogenation of a mixture of cycloadducts **264a–c** using 10% Pd/C (Scheme 73).

**Scheme 74:**
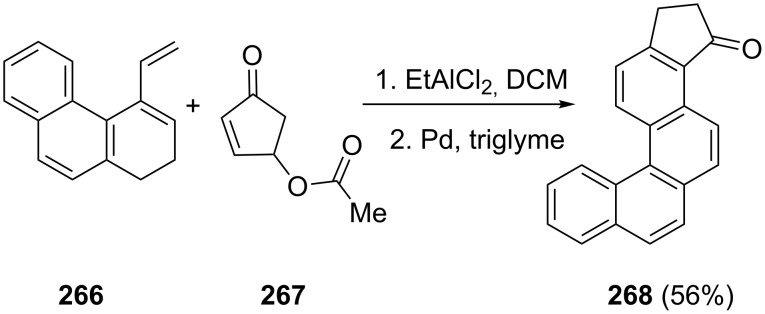
Synthesis of 1-indanone **268** from dihydro-3-vinylphenanthrene **266** and 4-acetoxy-2-cyclopenten-1-one (**267**).

An interesting example is the Diels–Alder reaction between dihydro-3-vinylphenanthrene (**266**) and 4-acetoxy-2-cyclo-penten-1-one (**267**) which led to formation of the helicene-like product **268** with the 1-indanone core (Scheme 74) [103].

**Scheme 75:**
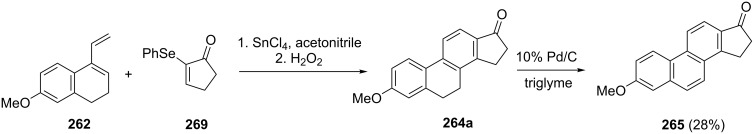
Synthesis of 1-indanone **271** from phenylselenyl-substituted cyclopentenone **268**.

The Diels–Alder reaction of **262** and phenylselenyl-substituted cyclopentenone **269** was less effective and gave 1-indanone **265** in 28% yield only (Scheme 75) [102]. Another example of this reaction catalyzed by a Lewis acid has also been reported [104].

**Scheme 76:**
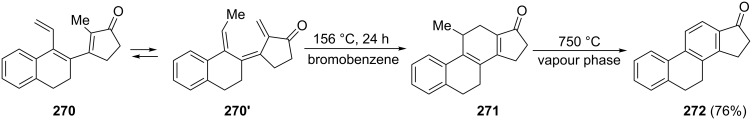
Synthesis of 1-indanone **272** from the trienone **270**.

The flash vacuum pyrolysis has been applied for aromatization of **271** to afford 1-indanone **272** in 76% yield. The former **271** was obtained from the trienone **270/270’** which underwent ring closure to give the 6-membered ring [105] (Scheme 76).

#### 2.2 From alkynes

**Scheme 77:**
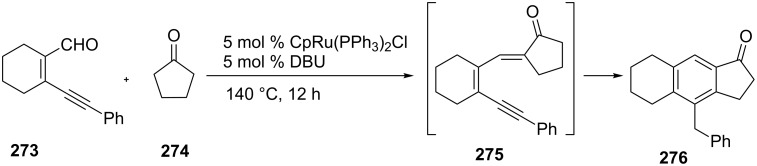
Synthesis of the 1-indanone **276** from the aldehyde **273**.

DBU and CpRu(PPh_3_)_2_Cl dual catalysts enabled a one-pot annulation of aldehyde **273** and cyclopentanone (**274**) to give the 1-indanone derivative **276** [106]. The new catalytic reaction which replaced a previously described four-step synthesis [107], involved a tandem aldol condensation/dehydration and cyclization of the intermediate **275** to **276** (Scheme 77).

**Scheme 78:**
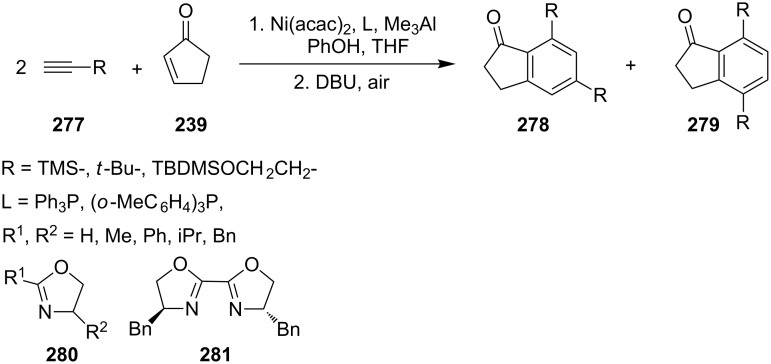
Synthesis of 1-indanones **278** and **279**.

In 1999, Ikeda and Mori have presented a cyclotrimerization of enones (e.g., cyclopentenone **239**) with alkynes in the presence of nickel and aluminum complexes [108]. This [2 + 2 + 2] cycloaddition run with a high regioselectivity and led mostly to *meta* isomers. The authors used, as catalytic systems, the following complexes: Ni(acac)_2_, Ni(cod)_2_, Me_3_Al, Me_2_Al(OPh), MeAl(OPh)_2_ and Al(OPh)_3_. In 2000, Ikeda and Kondo have continued their studies on regioselectivity of the cyclotrimerization [109] and investigated the effects of various ligands (L) on regioselectivity and yields of this reaction (Scheme 78). In case of application of triarylphosphines (Ph_3_P and (*o*-MeC_6_H_4_)_3_P) as ligands, only *para* isomers **279** were formed in moderate 33% and 49% yields, respectively. On the contrary, when oxazolines **280** or **281** were used as ligands, mainly *meta* isomers **278** were formed with high yields.

**Scheme 79:**
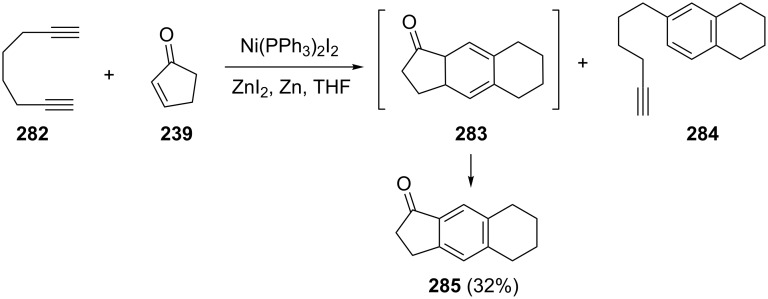
Synthesis of 1-indanone **285** from octa-1,7-diyne (**282**) and cyclopentenone **239**.

Cheng et al. have obtained 1-indanone **285** from octa-1,7-diyne (**282**) and cyclopentenone **239** as a result of Ni-complex-catalyzed [2 + 2 + 2] cyclotrimerization proceeding via the intermediate **283** [110] (Scheme 79). The dimer **284** of the starting dialkyne has also been obtained.

#### 2.3 From *o*-bis(dibromomethyl)benzene

**Scheme 80:**
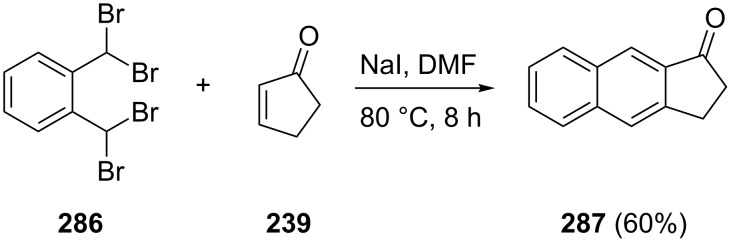
Synthesis of benz[*f*]indan-1-one (**287**) from cyclopentenone **239** and *o*-bis(dibromomethyl)benzene (**286**).

Erenler et al. have utilized *o*-bis(dibromomethyl)benzene (**286**) and cyclopentenone **239** to the synthesis of benz[*f*]indan-1-one (**287**) and its bromo derivative [111]. Both compounds are promising reagents for the synthesis of biologically active compounds (Scheme 80).

Kubo et al. have synthesized **287** from the same substrates **239** and **286** by a slight change of reaction conditions [112].

**Scheme 81:**
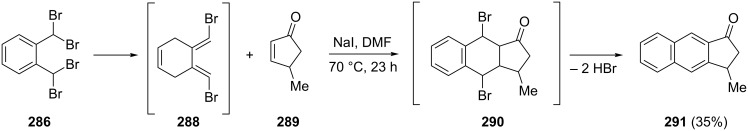
Synthesis of 3-methyl-substituted benz[*f*]indan-1-one **291** from *o*-bis(dibromomethyl)benzene (**286**) and 4-methylcyclopent-2-enone (**289**).

Jones et al. have synthesized 3-methyl-substituted benz[*f*]indan-1-one **291** in 35% yield from *o*-bis(dibromomethyl)benzene (**286**) and 4-methylcyclopent-2-enone (**289**) (Scheme 81) [113].

#### 2.4 From other compounds

**Scheme 82:**
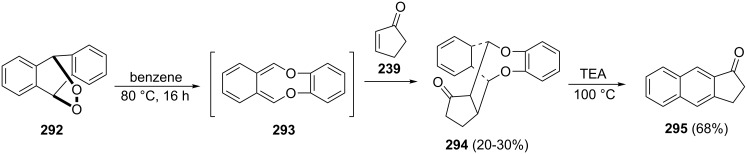
Synthesis of benz[*f*]indan-1-one (**295**) from the anthracene epidioxide **292**.

Albrecht, Defoin and Siret have synthesized benz[*f*]indan-1-one (**295**) from the anthracene epidioxide **292**, which underwent thermal isomerization to give the reactive intermediate **293** [114]. As a result of the Diels–Alder reaction of the latter with cyclopentenone **239**, the adduct **294** was formed, which was further subjected to the TEA-induced cleavage at 100 °C to give the desired 1-indanone **295** (Scheme 82).

**Scheme 83:**
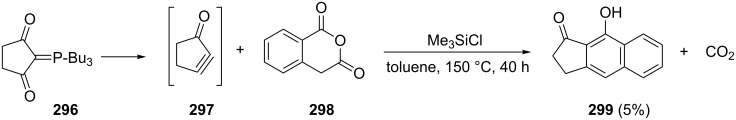
Synthesis of 1-indanone **299** from homophthalic anhydride **298** and cyclopentynone **297**.

Masui et al. have synthesized 1-indanone **299** in 5% as a result of the Diels–Alder reaction between homophthalic anhydride (**298**) and cyclopentynone **297** generated from the phosphorane **296** by the intramolecular Wittig reaction (Scheme 83) [115].

**Scheme 84:**
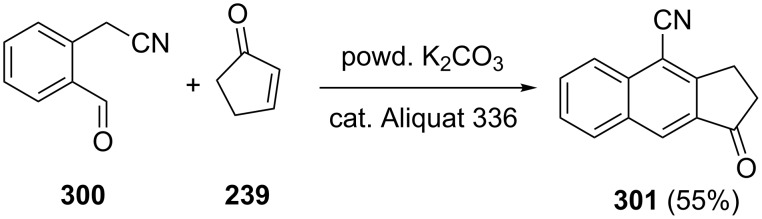
Synthesis of cyano-substituted 1-indanone derivative **301** from 2-cyanomethylbenzaldehyde (**300**) and cyclopentenone **239**.

Jończyk et al. have synthesized cyano-substituted 1-indanone derivative **301** in 55% yield under solid–liquid, phase-transfer catalysis conditions [116]. In this synthesis, 2-cyanomethylbenzaldehyde (**300**) was reacted with cyclopentenone **239** in the presence of powdered K_2_CO_3_ and Aliquat^®^ 336 as a catalyst. (Scheme 84).

### 3 Construction of the 5- and 6-membered rings

#### 3.1 From alkynes

**Scheme 85:**
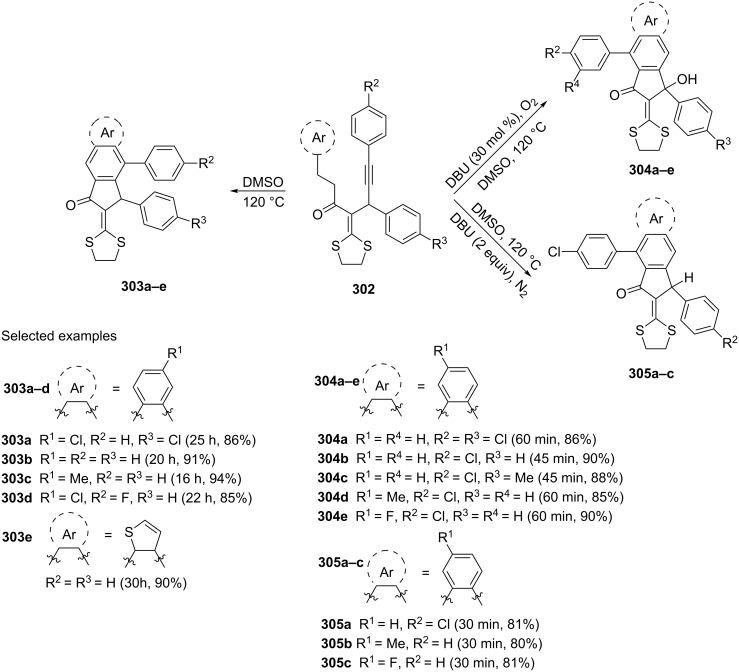
Synthesis of 1-indanone derivatives **303**–**305** from ketene dithioacetals **302**.

The intramolecular, dehydro-Diels–Alder reaction of ketene dithioacetals **302** leading to formation of various benzo[*f*]-1-indanones **303–305**, has been described in 2015 by Bi et al. [117]. Modulation on the reaction parameters such as addition of DBU and the type of atmospheric gas used (O_2_, N_2_), regulated the regioselective formation of the 1-indanones **303–305** (Scheme 85).

**Scheme 86:**
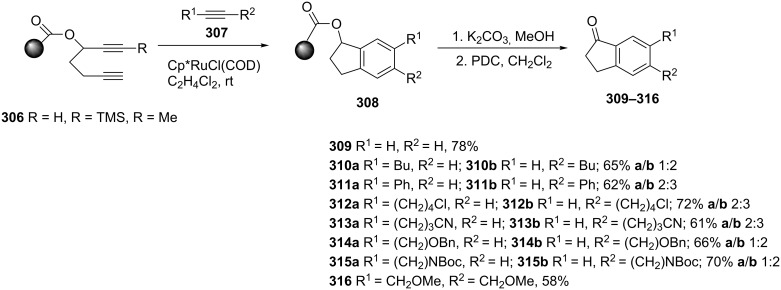
Synthesis of 1-indanones **309**–**316**.

A new, simple approach for the synthesis of natural and unnatural 1-indanones **309–316** has been proposed by Deiters et al. [118]. The key step of this synthesis was associated with [2 + 2 + 2] cyclotrimerization of the dialkyne **306** with variously disubstituted alkynes **307** performed on a solid phase TentaGel^®^ resin (0.25 mmol/g) in the presence of Ru catalyst (Scheme 86). In case of **309–316**, this reaction led to the formation of mixtures of two regioisomers. The examined regioisomeric ratios (**a**/**b**) were ranged from 1:2 to 2:3 with a preference to **310a**–**315a** regioisomers.

**Scheme 87:**
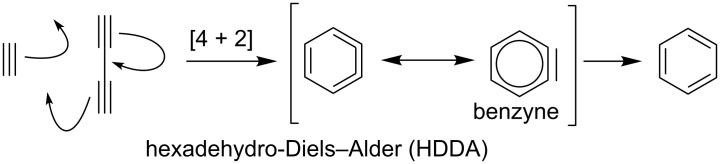
Mechanism of the hexadehydro-Diels–Alder (HDDA) reaction.

An interesting approach to the synthesis of 1-indanones and 1-indenones is based on the hexadehydro-Diels–Alder (HDDA) reaction in which an alkyne reacts in the [4 + 2] cycloaddition with diyne and forms a reactive benzyne species as a precursor of the benzene ring (Scheme 87).

**Scheme 88:**
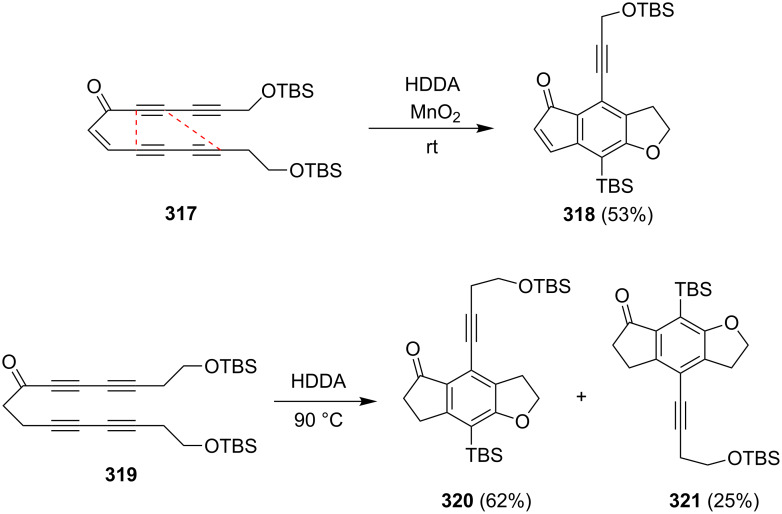
Synthesis of 1-indenone **318** and 1-indanones **320** and **321** from tetraynes **317** and **319**.

**Scheme 89:**
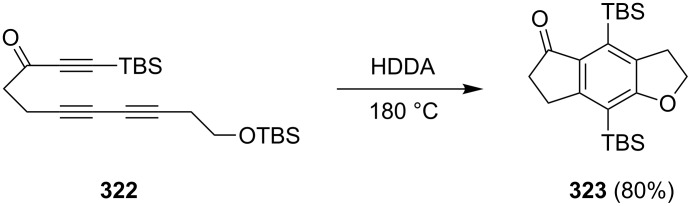
Synthesis of 1-indanone **320** from the triyn **319**.

This methodology has been applied in the synthesis of 1-indanones (Scheme 88 and Scheme 89).

In 2012, Hoye et al. have presented the synthesis of 1-indenone **318** via a hexadehydro-Diels–Alder (HDDA) reaction with simultaneous formation of five and six-membered rings from the tetrayne **317** (Scheme 88) [119]. In the reaction participates only three triple bonds marked by red lines. This, catalyzed by MnO_2_ reaction, is fully regioselective. During the cycloaddition after the formation of the five and six-membered rings, one of the *tert*-butyldimethylsilyl (TBS) group migrates from an oxygen to the triple bond of benzyne to give **318**. In 2014, the authors have shown that the HDDA cyclization of the unsymmetrical substituted ketotetrayne **319** gives a mixture of isomeric 1-indanones **320** and **321** (Scheme 88) [120]. It is the effect of competition between two modes of the cycloaddition reaction. In the “normal” mode of this reaction, cyclization takes place between the triple bond in α,β-position and the diyne in γ’,ε’-position to give **320**. In the “abnormal” mode, the cyclization takes places between the triple bond in γ’-position and the diyne in α,γ-position to give **321**.

Hoye et al. have further expanded the scope of this reaction on several other substrates which are active in the [2 + 2 + 2] cycloaddition [120]. For example, the triyn **322** under hexadehydro-Diels–Alder (HDDA) conditions gave the corresponding 1-indanone **323** in 80% yield (Scheme 89).

#### 3.2 From furans

**Scheme 90:**
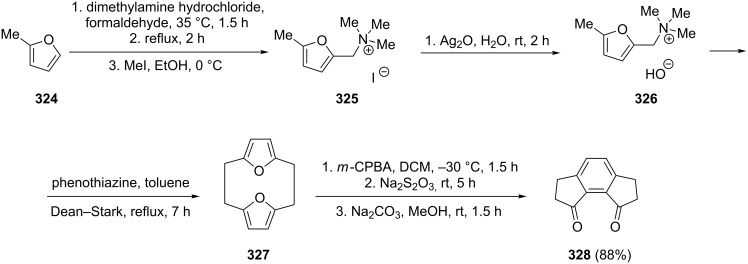
Synthesis 1-indanone **328** from 2-methylfuran **324**.

Van der Eycken et al. have synthesized 1-indanone **328** by utilizing 2-methylfuran (**324**) as a starting compound which was converted to the Mannich adduct **325**, followed by the anion exchange reaction to give ammonium hydroxide **326** [121]. The latter underwent dimerization to afford the furanocyclophane **327**, which was next oxidized with *meta*-chloroperoxybenzoic acid (*m*-CPBA), followed by a Diels–Alder reaction and dehydration to obtain 1-indanone **328** in 88% yield (Scheme 90).

**Scheme 91:**
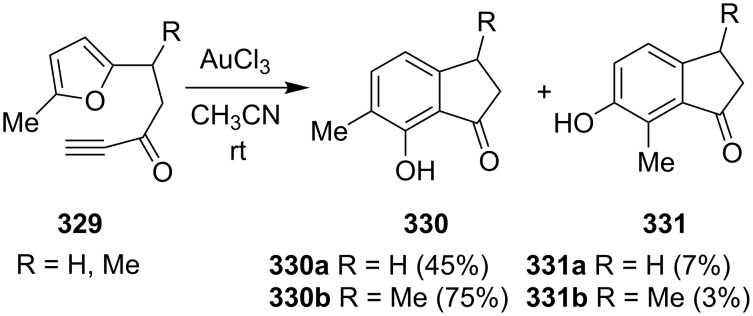
Synthesis of 1-indanones **330** and **331** from furans **329**.

In 2003, Hashmi et al. have demonstrated an intramolecular gold catalyzed [4 + 2] cycloaddition of furans **329** with a tethered alkyne moiety [122]. The reaction was regioselective and gave 1-indanones **330** at room temperature, in good yields up to 75%. The second regioisomer was formed only in 3–7% yield (Scheme 91). 7-Hydroxy-6-methylindan-1-one **330** has later been used in the synthesis of natural sesquiterpene, jungianol isolated from *Jungia malvaefolia*.

#### 3.3 From bicyclic compounds

**Scheme 92:**
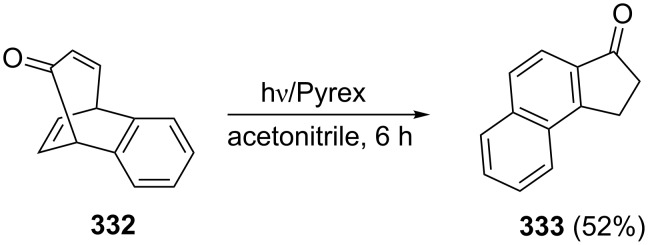
Synthesis of 1-indanone **333** from the cycloadduct **332**.

Ciabattoni, Crowley and Kende have obtained 1-indanone **333** in 52% yield by photoisomerization of benzobicyclo[3.2.2]nonatrienone **332** (Scheme 92) [123].

### 4 Functionalization of the 5- or 6-membered ring of 1-indanones or related compounds

Another approach to obtain bioactive 1-indanones is their functionalization. In this way, scientists have synthesized C5- and C6-alkoxy and benzyloxy-substituted 1-indanones by the alkylation reaction of 5-hydroxy-1-indanone or 6-hydroxy-1-indanone with various alkyl or benzyl bromides. These 1-indanone derivatives are potential inhibitors of two separate isoforms of monoamine oxidases: MAO-A and MAO-B [124]. Monoamine oxidases (MAO) are mitochondrial enzymes that catalyze two-electron oxidation of amine substrates. MAO terminates physiological actions of amine neurotransmitters in brain; therefore, MAO inhibitors have been applied in the treatment of neurodegenerative and neuropsychiatric disorders such as Parkinson’s disease and depression. The studies have shown that synthesized C6-substituted 1-indanones are effective and selective MAO-B inhibitors, while C5-substituted 1-indanones are less effective MAO-B inhibitors.

A 2,4-dinitrophenylhydrazone derivative of 1-indanone with a potent antimicrobial activity has been synthesized in 2014 by Obafemi et al. utilizing unsubstituted 1-indanone as a starting material [125]. This bioactive derivative has been obtained by Claisen–Schmidt reaction of 1-indanone with benzaldehyde, followed by condensation with 2,4-dinitrophenylhydrazine (DNP). This compound exhibited the best activity with the lowest MIC (minimum inhibitory concentration) values for four Gram-negative bacterial strains, such as: *Pseudomonas aeruginosa, Salmonella typhimurium, Shigella flexineri* and *Acinetobacter calcaoceuticus anitratus* (15.6 μg/mL), and two following Gram-positive bacterial strains: *Staphylococcus aureus* and *Micrococcus luteus* (31.3 μg/mL).

**Scheme 93:**
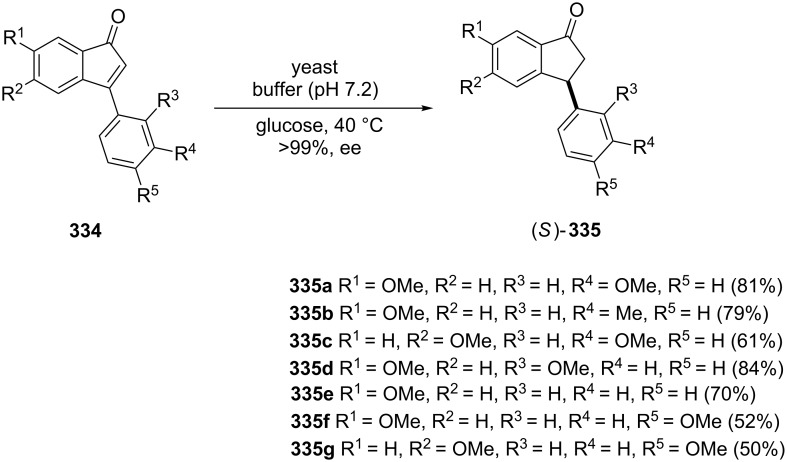
Synthesis of (*S*)-3-arylindan-1-ones **335**.

The reduction of 1-indenones to 1-indanones has been applied by Clark et al*.* [126]. The authors used bakers’ yeast (*Saccharomyces cerevisiae*) for the reduction of 3-arylinden-1-ones **334** to obtain (*S*)-3-arylindan-1-ones **335** with high enantioselectivity (Scheme 93).

Methyl *N*-benzyl-4-methylpiperidinecarboxylate acylation with 5,6-dimethoxy-1-indanone has been applied as the key step of the synthesis of 2-((1-benzyl-4-piperidinyl)hydroxymethyl)-5,6-dimethoxy-1-indanone [127].

**Scheme 94:**
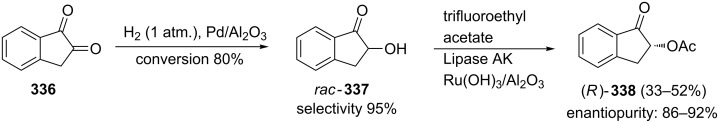
Synthesis of (*R*)-2-acetoxy-1-indanone **338**.

Regioselective hydrogenation of the diketone **336** followed by chemoenzymatic, dynamic kinetic resolution of the resulting *rac*-2-hydroxy-1-indanone (**337**) has been used for the synthesis of (*R*)-2-acetoxy-1-indanone (**338**) (Scheme 94) [128].

1-Indanones may also be synthesized by oxidation of the 1-indane core. For example, scientists have oxidized unsubstituted indane to 1-indanone by using a metal-free catalytic system consisting of aryl-tetrahalogenated *N*-hydroxyphthalimides (TCNHPI) and 1,4-diamino-2,3-dichloroanthraquinone (DADCAQ) in very good yield (98%) [129]. Another catalyst, applied for the synthesis of 1-indanone, is mesoporous Mn_0.5_Ce_0.5_O_x_ which allows a selective oxidation of hydrocarbons under mild conditions [130]. This compound catalyzed the oxidation of unsubstituted indane in which 1-indanone was obtained as the main product along with 1-indanol. An efficient synthesis using microreactors for oxidation of benzylic compounds such as xanthene, fluorene, 3,4-dihydro-2*H*-naphthalene, indane and diphenylmethane has been proposed by Jia et al. [131]. Thus, indane was oxidized to 1-indanone in high yield (94%) by oxygen formed in the reaction of NaClO with *tert*-butyl hydroperoxide.

2,3-Dichloro-5,6-dicyano-1,4-benzoquinone (DDQ) has also been used to oxidize the indane core [132]. This reaction constituted the first step in the synthesis of hydroxy-substituted, carcinogenic cyclopenta[α]phenanthrene.

**Figure 6:**
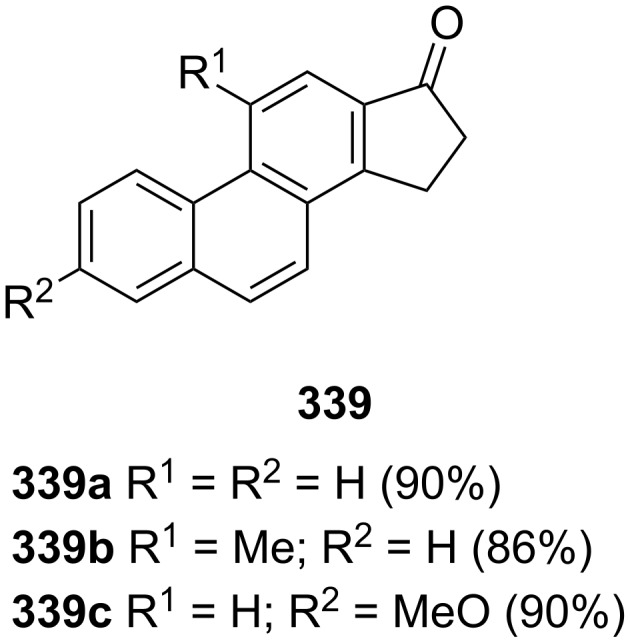
Chemical structures of obtained cyclopenta[α]phenanthrenes **339**.

Harvey and Lee have reported a synthesis of carcinogenic cyclopenta[*α*]phenanthrenes containing a 1-indanone core **339** [133] (Figure 6).

Other catalysts applied for the oxidation of indanes or 1-indanols to 1-indanones are ruthenium catalysts immobilized on a solid phase. Thus, a poly(ethylene glycol)-supported ruthenium complex catalyzed oxidation of indane to 1-indanone in 99% yields [134]. SiO_2_-supported iodoarene–RuCl_3_ bifunctional catalyst catalyzed the conversion of indane into 1-indanone in 92% yield [135].

1-Indanol tricarbonylchromiums have been oxidized with MnO_2_ to optically pure 1-indanone tricarbonylchromium derivatives by Jaouen and Meyer [136].

**Scheme 95:**
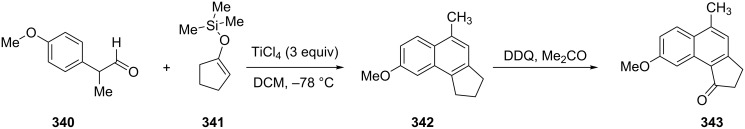
Synthesis of the benzoindanone **343** from arylacetaldehyde **340** with 1-trimethylsilyloxycyclopentene (**341**).

Voskoboynikov et al. have also applied the oxidation of the indane core to obtain benzoindanone **343** [137]. As a result of the TiCl_4_-catalyzed reaction of arylacetaldehyde **340** with 1-trimethylsilyloxycyclopentene (**341**), cyclopenta[α]naphthalene **342** was formed (Scheme 95) [137]. The latter was then oxidized to the benzoindanone **341** with dichlorodicyanobenzoquinone (DDQ).

## Conclusion

This article is the first comprehensive work reviewing original and patent literature of synthetic methods and biological applications of 1-indanones. It has been shown that these bioactive molecules may be obtained from a variety of starting materials. The commonly used reactions leading to the formation of the title compounds are Nazarov, Knoevenagel, Diels–Alder, and Friedel–Crafts alkylation and acylation reactions. The structural diversity of 1-indanones implies various biological responses and these compounds may be applied in agriculture and medicine. Some of the 1-indanone derivatives may constitute a new hope, as future drugs, for the patients suffering from Alzheimer’s and Parkinson’s diseases, and those infected with hepatitis C virus. Single applications for organic optoelectronics have also been reported. Due to the wide application potential, 1-indanones are interesting objects for further investigations and it is desirable to design new methods for their synthesis.
